# A Spectroelectrochemical Study of the Effect of Asymmetry on the Electrochemical Response of Lipid Bilayers

**DOI:** 10.1021/acs.jpcb.5c05655

**Published:** 2026-03-10

**Authors:** Elena Madrid, Sarah L. Horswell

**Affiliations:** School of Chemistry, 1724 University of Birmingham, Edgbaston, Birmingham B15 2TT, U.K.

## Abstract

The effect of asymmetry in supported lipid bilayers on their electrochemical phase behavior has been studied using in situ Polarization Modulation Infrared Reflection Absorption Spectroscopy (PM-IRRAS). Dimyristoylphosphatidylcholine (DMPC) and dimyristoylphosphatidylethanolamine (DMPE), which have the same tails and different headgroups, have been used to construct asymmetric bilayers on Au(111) electrodes. The organization and orientation of the hydrocarbon tails in each leaflet of the asymmetric bilayers have been characterized separately by deuterating the tails in the opposing leaflet. The vibrational frequencies of the chain methylene stretching modes show that DMPC is relatively ordered in asymmetric bilayers, and DMPE is relatively disordered, compared with their respective symmetric bilayers. The tail orientations in the as-deposited asymmetric bilayers are similar, showing the two monolayers influence each other, but the changes induced in each bilayer by the application of a potential difference across the bilayer are different and indicate that the bilayers decouple, with each monolayer responding separately to the imposed field. The results suggest that the behavior of previously reported symmetric systems may be more strongly influenced by the properties of the electrolyte-facing leaflet and highlight the value of using electrochemical perturbation of lipid bilayers in structural studies to provide additional insights into lipid–lipid interactions.

## Graphical abstract

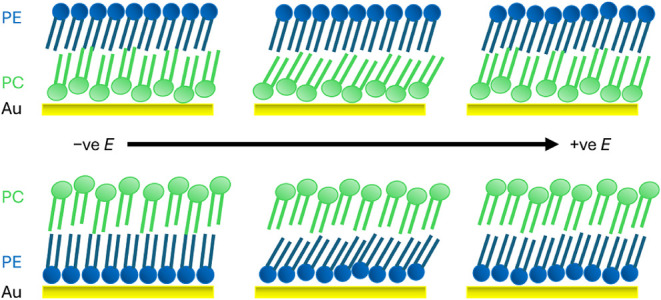

## 1 Introduction

Biological cell membranes perform a range of functions, including acting as a selective barrier for the cell, organelles or for transport, and allowing the cell to communicate with and respond to its environment.^1,2^ They are based on a bilayer of amphiphilic lipid molecules containing a range of proteins. Lipid bilayers have thus been a popular model for biological cell membranes, as they allow a more natural environment in which to study protein function or to host receptors for sensors.^3–5^ In more fundamental studies, they can be used to reduce the complexity and variability of the system, enabling researchers to control the parameter space and develop an understanding of the interactions between different experimental parameters and membrane function.^6–8^ Common lipid models include vesicles, lipid monolayers at the air/water interface, unsupported bilayers and supported, tethered, or floating monolayers or bilayers (including hybrid films).^7,9^ Each model has its own distinct advantages; for example, vesicles may mimic the natural curvature of a cell membrane, monolayer studies allow control over the membrane tension and unsupported bilayers offer the ability to monitor passage through the bilayer electrochemically. Bilayers based on surfaces enable a wide range of surface-sensitive tools to be used to bring together information on bilayer thickness, topography and mechanical properties,^10–14^ bilayer thickness and degree of solvation,^15–17^ and molecular organization, packing, and orientation.^18,19^ If a monolayer or bilayer is supported on an electrode surface, a tunable electric field can be applied across the bilayer to investigate electrochemical barrier properties and phase behavior.^5,20–26^ Surface-sensitive tools can used to determine how the bilayers behave under the influence of electric fields similar to those found in natural systems^25–29^ and how the bilayers interact with peptides.^30–34^

Many of the model systems employed are symmetric in composition; symmetric systems are easier to produce and often the focus of the study may not warrant the additional complexity of generating an asymmetric bilayer. Yet natural cell membranes are asymmetric and this asymmetry can have important consequences.^35–38^ For example, phosphatidylserine (PS) is normally maintained in the cytosolic leaflet of the mammalian plasma cell membrane and its presence in the outer leaflet acts as a signal to induce apoptosis and the death of the cell.^39,40^ Some tumor cells also express PS on the outer surface, which can result in their recognition by monocytes.^41,42^ Asymmetry in mimics of mitochondrial membranes has been shown to affect surface charge, membrane-bound protein concentration, and susceptibility to permeabilization.^43^ Asymmetry is also believed to be important for vesicle trafficking and amphipath transport,^44^ endocytosis,^45^ membrane curvature^46,47^ and cell development, differentiation, and division.^48,49^ Cells employ enzymes (scramblase, flippase, and floppase) to control carefully this asymmetry.^1,44^ The ease or difficulty of transfer of lipids between the two monolayers (“flip-flop”) has attracted interest, with sum frequency generation (SFG) being employed to determine the extent and rate of transfer.^50–53^ These studies showed fluidity and temperature to be important parameters. Neutron reflectometry investigations have shown that only lipids in their fluid phases undergo significant transfer between monolayers^54^ and in other cases the membrane asymmetry can be maintained for many hours.^54,55^ Asymmetry in vesicles affects bilayer mechanical properties^56,57^ and the formation of ordered domains.^58,59^ Depending on the composition of the two leaflets, one can exert an influence on the structure and behavior of the other or the two can behave independently.^58–60^ It has been shown that curvature and stress in membranes is related to the distribution of cholesterol, with a fine balance between thermodynamic effects and stress.^61–63^ Simulations have shown that the distribution of cholesterol depends quantitatively on the difference in order between lipids in the two halves of the bilayer.^64^ The formation of ordered domains (rafts) in one leaflet of an asymmetric bilayer can induce ordering directly opposite in the other leaflet, depending on the melting temperature and/or chain length of the lipids in that leaflet,^65–67^ and sometimes even if the opposing bilayer is strongly predisposed to disorder, potentially via local redistribution of cholesterol.^68^ However, theoretical studies have also shown that domains in opposing leaflets can be either correlated with each other or in antiregistration, depending on chain length.^69^

To study electrochemical properties and electric field-induced phase behavior in asymmetric bilayers, it is convenient to deposit lipid monolayers sequentially using Langmuir–Blodgett (LB) and Langmuir–Schaefer (LS) methods. This method of deposition has been used to study the two monolayers independently, using isotopic substitution to separate their spectroscopic signals.^70,71^ Electrochemists have compared the barrier properties obtained,^72,73^ have used different composition in the leaflets to facilitate a particular architecture^74^ or have used asymmetric bilayers to enable the study of lipids that do not transfer well via LB deposition, using one lipid as a base to deposit the second.^75^ However, there appear to be few studies aimed at characterizing the effect of chemical asymmetry on the electrochemical phase behavior.^73^ A study of asymmetric bilayers built from dimyristoylphosphatidylcholine (DMPC), dimyristoylphosphatidylethanolamine (DMPE) and dimyristoylphosphatidylserine (DMPS) showed the main differences in electrochemical properties to be related to the location of the anionic lipids. The bilayers built from DMPE and DMPC exhibited similar equilibrium behavior but the differential capacitance was lower in bilayers where DMPE was adjacent to the electrode.^73^ The aim of the present study is to establish whether this means Au|DMPE|DMPC and Au|DMPC|DMPE bilayers have the same or different structure and whether the two leaflets of the bilayer alter one another’s structural response to an electric field. These bilayers represent a simple model for the plasma cell membrane, which is abundant in phosphatidylcholines (PC) in the outer leaflet and phosphatidylethanolamines (PE) in the cytosolic leaflet.^1,44^ This choice also utilizes two lipids for which data corresponding to their symmetric bilayers are available for comparison: DMPC^27,70^ and DMPE.^71,76^ The structures of these lipids are given in Figure 1. The lipids have the same tail length and different headgroups. The DMPE headgroup is smaller than the DMPC headgroup and is capable of intermolecular hydrogen bonding, which has a strong influence on its packing and phase behavior: saturated PE lipids have significantly higher chain-melting phase transition temperatures than saturated PC lipids.^77^ By deuterating one of these lipids at a time, we are able to follow the field-driven changes in the undeuterated monolayer alone using in situ Polarization Modulation Infrared Reflection Absorption Spectroscopy (PM-IRRAS). We show that the DMPE and DMPC in opposing monolayers influence one another’s ordering but do not behave as a coupled bilayer upon perturbation by the field. Instead, the two leaflets exhibit distinct responses to the field that, in turn, enable us to deduce that the behavior of symmetric bilayers is determined by the properties of the electrolyte-facing leaflet. This finding suggests that investigating asymmetric lipid bilayers with in situ IR spectroscopy can also provide insight into the electrochemical behavior of symmetric systems.

**Figure 1:**
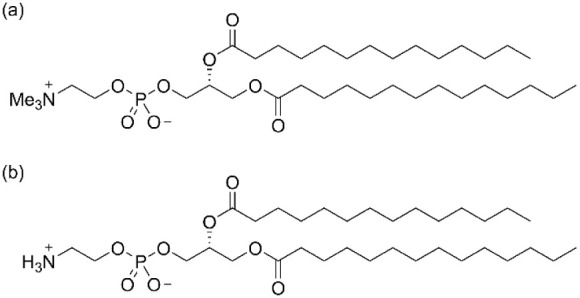
Chemical structures of (a) DMPC and (b) DMPE.

## 2 Experimental Section

### 2.1 Materials and Cleaning

Ultrapure water (resistivity >18 MΩ cm, TOC < 5 ppb) was used throughout and was obtained from a tandem Milli-Q Elix-Gradient A10 system (Millipore, France). The electrolyte for PM-IRRAS experiments was prepared from sodium fluoride (Puratronic, 99.995% metals basis, Alfa Aesar, UK) and deuterium oxide (Sigma-Aldrich) at a concentration of 0.1 M. DMPE, DMPC, and their chain-perdeuterated analogues (D54-DMPE and D54-DMPC, respectively) were obtained from Avanti Polar Lipids (Birmingham, AL) and used without further purification. DMPC (*h*-DMPC) and D54-DMPC (*d*-DMPC) solutions were prepared in chloroform (HPLC grade, Sigma-Aldrich). DMPE (*h*-DMPE) and D54-DMPE (*d*-DMPE) solutions were prepared in a 9:1 v/v mixture of chloroform and methanol (HPLC grade, Sigma-Aldrich).

Volumetric glassware was cleaned with piranha solution (Caution! This is a highly exothermic process that may cause an explosion!), followed by thorough rinsing with ultrapure water. The glassware was soaked overnight in ultrapure water and rinsed again directly before use. Glassware used for preparing lipid solutions was then rinsed with methanol, the chloroform/methanol mixture and then chloroform, to remove water. All other glassware was prepared by heating in a 50:50 mixture of concentrated nitric and sulfuric acids for ∼1 h, then rinsing with copious amounts of ultrapure water and soaking overnight in ultrapure water. The glassware was again rinsed directly before use or dried in a designated clean oven. The PTFE, Kel-F, and PFA parts of the spectroelectrochemical cell were cleaned by soaking in a 50:50 mixture of 30% hydrogen peroxide solution and 30% ammonia solution for several hours, followed by thorough rinsing with ultrapure water and soaking overnight in ultrapure water. They were then rinsed again with ultrapure water and dried in a designated clean oven.

### 2.2 Bilayer Preparation

Langmuir–Blodgett (LB) deposition followed by Langmuir–Schaefer (LS) deposition was used to prepare asymmetric Y-type bilayers on a Au(111) substrate. A large area (600 cm^2^) Langmuir–Blodgett 611 trough (Nima, UK) equipped with a Delrin barrier and a dipping mechanism was used for the depositions. The surface pressure was monitored with a paper Wilhelmy plate and the subphase temperature was maintained at 19 °C, to match the conditions used in the infrared measurements. The trough was cleaned with the 9:1 v/v chloroform:methanol mixture and filled with ultrapure water. The cleanliness of the water was verified before measurements by closing the barrier and checking the surface pressure remained at zero. The Au(111) sample was flame-annealed as described previously^76^ and lowered into the subphase. The water cleanliness was checked again and lipid solution (typically ∼50 μL) was deposited dropwise across the surface of the water, using a Hamilton syringe. After equilibration (∼10 min), a pressure–area isotherm was recorded with a barrier speed of 25 cm^2^ min^–1^ and a target pressure of 40 mN m^–1^ for DMPC or 47 mN m^–1^ for DMPE. The Au substrate was raised through the lipid monolayer at a rate of 2 mm min^–1^ with the target pressure maintained (LB deposition). The sample was dried in argon for 30 min. During this time, the monolayer on the trough was removed and the trough cleaned and prepared with a fresh monolayer of the other lipid. The new monolayer was closed to the desired target pressure and the now horizontal Au sample was lowered onto the surface at a speed of 2 mm min^–1^ and raised again at the same speed (LS or horizontal touch deposition). The sample was dried in argon for 30 min and assembled into the spectroelectrochemical cell.

### 2.3 PM-IRRAS Measurements

A three-electrode spectroelectrochemical cell was used for the PM-IRRAS measurements. The working electrode was an 8 mm diameter Au(111) single crystal, oriented to better than 0.5° (MaTecK GmbH, Germany). It was mounted in a Kel-F holder and connected to the outside of the cell with a Au wire. The counter electrode was a gold wire (99.995%, Alfa Aesar) wound around the inside of the cell to form a ring concentric to the working electrode. The reference electrode was a Ag|AgCl|3 M KCl (BASi, U.S.) and was housed in a compartment connected to the cell with a tube that served as a Luggin capillary. Potentials in this work are reported with respect to this Ag|AgCl reference electrode. The electrolyte was 0.1 M NaF in D_2_O. Sodium fluoride was chosen to suppress solubility of the window and D_2_O was chosen to shift water absorption to a spectral region away from the C–H stretching region. Sodium fluoride is also a nonadsorbing electrolyte, which does not disrupt the adsorption of the lipid bilayer. The electrolyte was deoxygenated by bubbling with argon for at least 45 min before use. The IR window was a BaF_2_ 1 in. equilateral prism (Crystran, UK) and was cleaned in an ozone chamber prior to assembly of the cell. Transmission spectra of DMPC vesicles were also measured to enable the calculation of their isotropic optical constants, which are needed for the quantitative analysis of PM-IRRA spectra;^78,79^ the optical constants for DMPE were taken from a previous publication.^76^ The transmission spectra were measured for a 0.675% v/v solution of DMPC in 0.1 M NaF solution in D_2_O in a transmission cell, which consisted of two BaF_2_ windows separated with a 10 μm spacer. The optical constants were calculated from the transmission spectra using software kindly provided by Dr V. Zamlynny (Acadia University, Canada).^80^

A Bruker Vertex 80v spectrometer was employed for infrared measurements. It was equipped with a liquid nitrogen-cooled MCT detector and an external PMA module that comprised a photoelastic modulator (PEM) with a ZnSe 50 kHz optical head (Hinds Instruments, U.S.) and a synchronous sampling demodulator (GWC Technologies, U.S.) to demodulate the signal. Spectra were acquired over 8000 scans and at a resolution of 2 cm^–1^.

The PEM was set to a half-wave retardation of 2900 cm^–1^ and the angle of incidence was set to 51°. Background spectra were recorded in the dry cell, to correct for the response of the PEM^78,79^ The cell was then filled with electrolyte and the position of the electrode with respect to the window adjusted with a micrometer screw until an electrolyte thickness of close to 2 μm was achieved. The thickness was determined by comparing the reflectivity with theoretical curves calculated using Fresnel 1 software, kindly provided by Dr V. Zamlynny (Acadia University, Canada).^80^ The angle of incidence and electrolyte thickness were chosen from values reported by Jackson and Zamlynny to achieve optimum signal.^81^ PM-IRRA spectra were then recorded at 19 °C (±1 °C) at a series of potentials, stepping the potential in the cathodic direction, to match our previous measurements for DMPE bilayers.^71,76^ This temperature is below the main chain-melting phase transition of each of the lipids (see below), to allow the comparison of chain tilt angles from the IR spectra with those from previously reported symmetric bilayers.^27,70,71,76^ The introduction of *gauche* conformers upon chain melting would lead to a change in apparent tilt angle because of the partial randomization of transition dipole orientations, so the tilt angles in the liquid crystalline phase would be approximate values^78^ and not comparable with the data for the corresponding symmetric bilayers.

## 3 Results

The electrochemical responses of the chemically asymmetric bilayers investigated in the present work will be compared below with the electrochemical responses of chemically symmetric bilayers in previous studies, both isotopically symmetric and isotopically asymmetric. Figure 2 illustrates the different bilayer types prepared in this and previous studies and defines the acronyms used in the presentation and discussion of the results.

**Figure 2:**
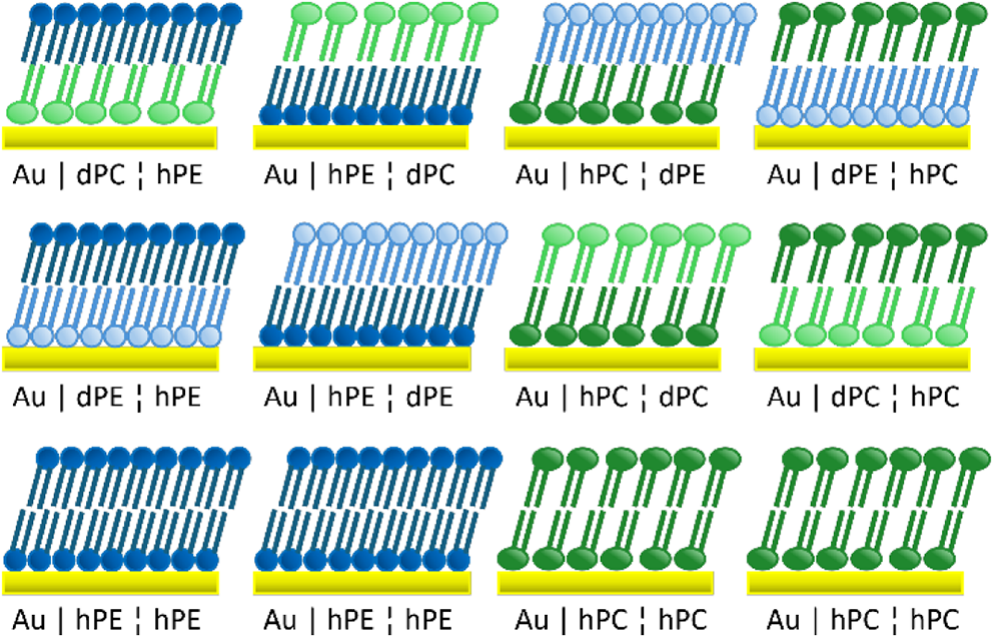
Schematic representations of the different bilayers discussed. DMPC is depicted with slightly larger headgroups than DMPE and deuterated lipids are represented in paler colors than their undeuterated counterparts. The top row of bilayers are chemically (and isotopically) asymmetric systems. The second row shows chemically symmetric but isotopically asymmetric systems investigated in refs 71 (PE) and 70 (PC). The bottom row represents chemically and isotopically symmetric systems reported in refs 76 (PE) and 27 (PC). In the text, comparisons are made both between bilayers containing the same lipid in different locations (the pairs in the top row) and between different bilayers containing a given lipid in the same location (bilayers within each column).

Figure 3 shows selected spectra acquired at different applied potentials. The left two panels show C–H stretching modes for DMPE (Au|dPC|**hPE** and Au|**hPE**|dPC) and the right two panels show C–H stretching modes for DMPC (Au|dPE|**hPC** and Au|**hPC**|dPE). The spectra for *h*-lipid next to the Au surface are given in the bottom two panels and the spectra for *h*-lipid adjacent to the electrolyte are given in the top two panels. The bands at ∼2958 cm^–1^ and ∼2873 cm^–1^ correspond respectively to the asymmetric and symmetric stretching modes of the methyl (CH_3_) groups.^77,82–88^ The peaks at ∼2919 cm^–1^ and ∼2852 cm^–1^ correspond respectively to the asymmetric and symmetric stretches of the methylene (CH_2_) groups.^77,83–88^ The atomic displacements and corresponding transition dipole moments associated with these two vibrations are illustrated schematically in Figure 4. Two Fermi resonances (FR), which arise from overlap of bending mode overtones with the methylene and methyl symmetric stretching vibrations,^82,88–90^ are also present in the spectra and so the spectra were fitted to six peaks as described in previous works.^27,70,71,76^ A Voigt line shape was used and an example fit is provided in Figure 4.^91^

**Figure 3:**
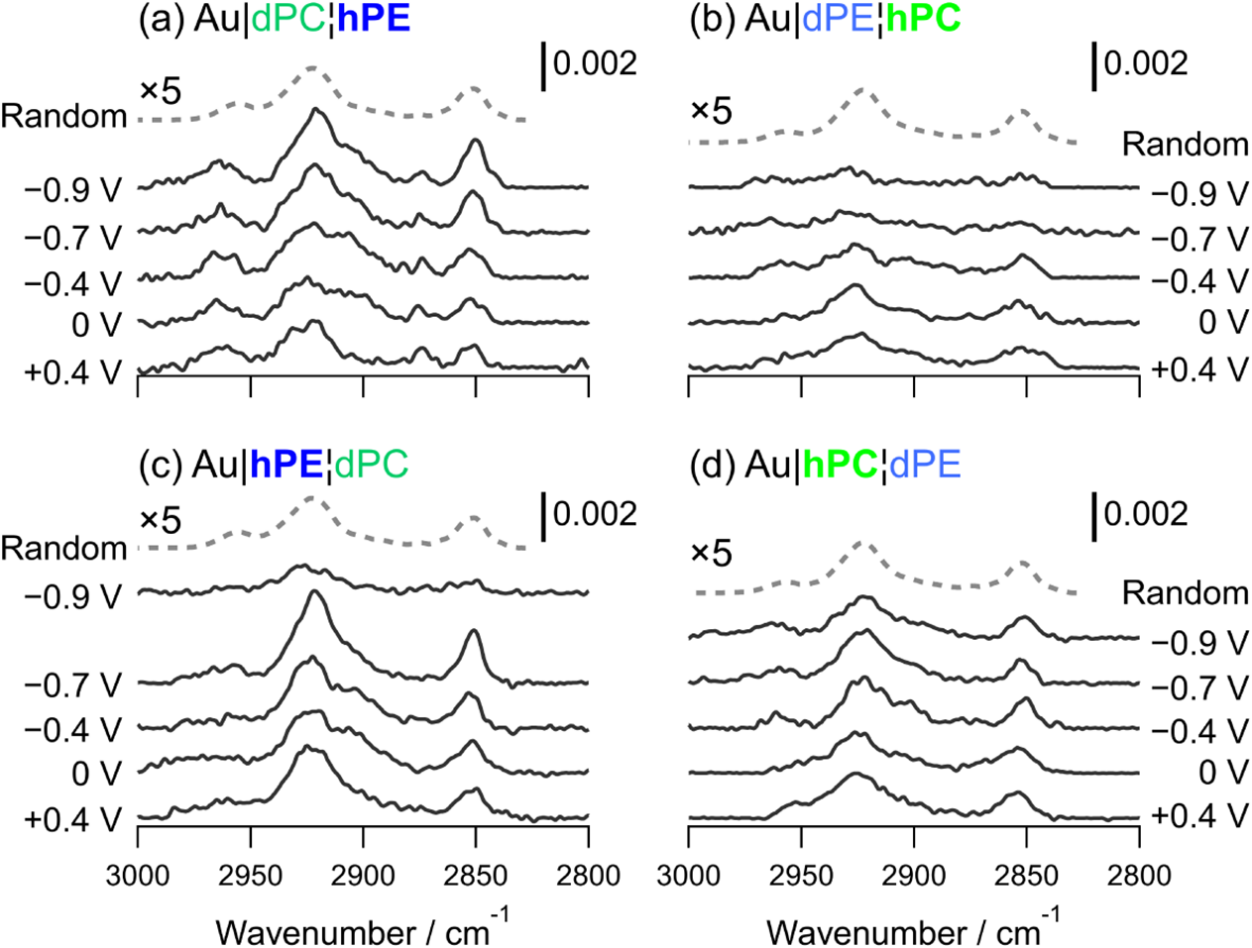
Selected PM-IRRA spectra in the C–H stretching region measured at the indicated applied potentials for asymmetric lipid bilayers transferred onto Au(111). (a) and (b) compare the two electrolyte-facing layers; (c) and (d) compare the two electrode-facing layers. (a) and (c) compare *h*-DMPE adjacent to the electrolyte (a) and adjacent to the electrode (c). (b) and (d) compare *h*-DMPC adjacent to the electrolyte (b) and adjacent to the electrode (d). The electrolyte was 0.1 M NaF in D_2_O. The spectra are offset by fixed amounts for clarity. The dashed lines are the theoretical spectra of randomly oriented molecules, scaled and offset for clarity.

The peak positions of the methylene stretching modes provide qualitative information on the organization of the hydrocarbon chains, while the full widths at half-maximum (FWHM) provide information on mobility of the lipids.^27^ The average wavenumbers and FWHM of the methylene stretching mode vibrations in Figure 3 are compared with reported values for symmetric bilayers in Table 1. Low wavenumbers indicate a small proportion of *gauche* conformers. On increasing the temperature, lipids undergo a chain-melting phase transition from a gel-like state or a ripple phase to a liquid crystalline state, which results in an increase in the average number of *gauche* conformers and increase in wavenumber.^77,82,83,86,87^ The phase transition for DMPC is at approximately 24 °C (23.6 °C in ref 77, 24.22 °C in H_2_O and 24.46 °C in D_2_O in ref 91) and the phase transition for DMPE is at approximately 50 °C (49 °C in ref 77 and 50.4 °C in ref 86). The phase transition temperatures of phosphocholines are lowered by 4–5 °C on deuteration,^92^ which could result in some disordering in the deuterated DMPC monolayers. To mitigate for this, the experiments were performed at 19 °C, which is also below the phase transition of *d*-DMPC. At the temperature used in the present work, DMPC is in the ripple phase and DMPE is in the gel phase. The wavenumbers of the methylene stretching modes are consistent with those expected for these phases.^77,82,83,86,87^

**Table 1:**
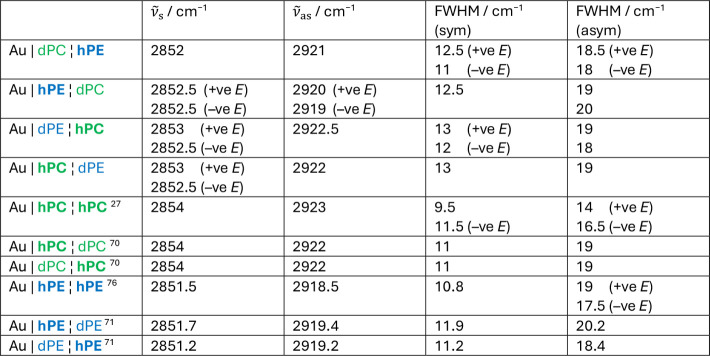
Fitted CH_2_ Symmetric and Asymmetric Stretching Mode Peak Positions and Full Widths at Half-Maximum Compared with Literature Data for Symmetric Bilayers^a^

a An error of around 1 cm^–1^ in position is estimated.

The integrated peak area can be used to calculate the orientation of the transition dipole moment associated with the vibration.^27,78,79^ The relationship between peak area and the angle between the transition dipole and the surface normal is given by eq 1:

1 $$\int Ad\mathrm{\nu ̃}\propto {|E\cdot \mu |}^{2}={|\mu |}^{2}\left\langle E^{2}\right\rangle \cos^{2}\theta$$

where *A* is the absorbance, *ṽ* is the wavenumber, μ is the dipole moment, ***E*** is the electric field vector and *θ* is the angle between the vector ***μ*** and the normal to the surface.^27,78,79^ The peak area also depends on the amount of material through which the beam passes. This can be accounted for by calculating the theoretical spectrum of randomly oriented molecules with the same cell geometry (angle of incidence, electrolyte thickness) and the optical constants of the materials involved. The tilt angle of the dipole moment may be obtained using eq 2:

2 $$\cos^{2}\theta =\frac{1}{3}\frac{\int_{E}Ad\mathrm{\nu ̃}}{\int_{random}Ad\mathrm{\nu ̃}}$$

and the peak areas in the experimental spectrum and in the theoretical spectrum.^27,78,79^ The transition dipole moment directions are orthogonal to one another and to the direction of the chain backbone (Figure 4). The tilt angle between the chain and the surface normal can be calculated using eq 3:^27,78,79,93^

3 $$\cos^{2}\theta_{\mathrm{as}}+\cos^{2}\theta_{s}+\cos^{2}\theta_{\mathrm{chain}}=1$$

**Figure 4:**
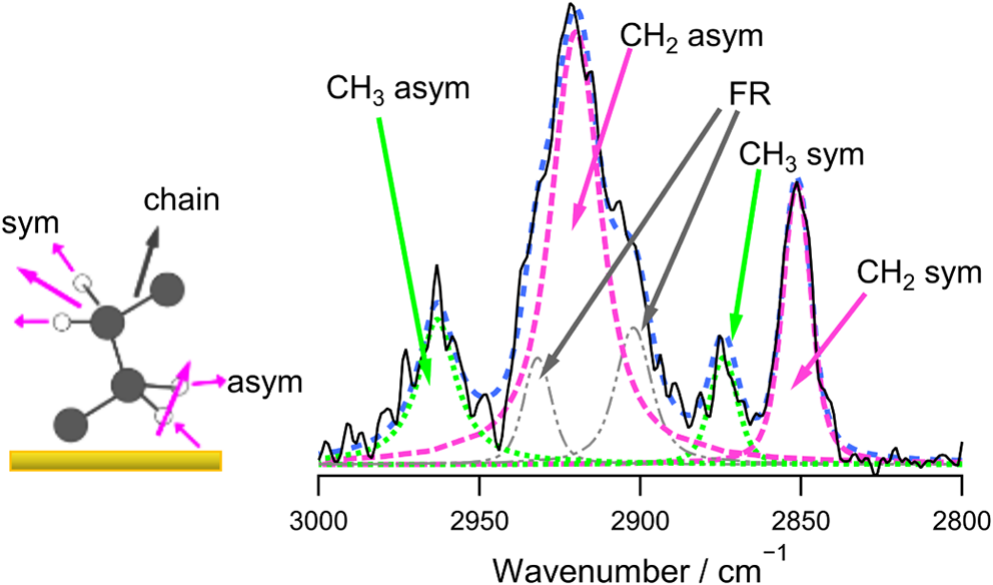
Left: cartoon showing the methylene stretching vibrations. The thicker arrows show the directions of the transition dipoles. Right: example of fitting peaks to a spectrum: Au | dPC | **hPE** at −0.7 V. FR stands for Fermi resonance.

Equations 2 and 3 mean that an increase in the integrated peak areas of the methylene stretching modes indicates an increase in the tilt angle of the hydrocarbon chain. Therefore, Figure 3c,d show that the chain tilt angle for the electrode-facing monolayer increases as the potential is made more negative but then decreases at the most negative potential, while the electrolyte-facing monolayers (Figure 3a,b) respond differently to the change in potential.

Figure 5 provides plots of the chain tilt angles against applied potential for each bilayer. The top panels compare how DMPE behaves in each leaflet, and how DMPC behaves in each leaflet. The bottom panels compare the inner, electrode-facing leaflets (Au|**hPE**|dPC and Au|**hPC**|dPE) and the outer, electrolyte-facing leaflets (Au|dPC|**hPE** and Au|dPE|**hPC**). Figure 5 shows that the location of the lipid in an asymmetric bilayer affects its response to the applied electric field. DMPE in the inner leaflet has a different dependence on potential from DMPE in the electrolyte-facing leaflet and, similarly, DMPC’s dependence on potential is different in each location. The behavior of DMPE in the electrolyte-facing leaflet is similar to that of undeuterated DMPE bilayers: relatively featureless but with a slight tendency to increase as the potential is made more negative.^76^ The behavior of DMPC in the electrolyte-facing leaflet resembles that of undeuterated DMPC bilayers: a small increase in tilt angle from positive potentials toward the phase transition (resulting from electrostriction) and a subsequent decrease at negative potentials.^27^ For DMPC bilayers, this decrease has been attributed to a change in organization of DMPC molecules on detachment of the bilayer from the surface, in which headgroups stagger, allowing closer packing of chains with a lower tilt angle.^27^ The main difference between *h*-DMPC on *d*-DMPE and *h*-DMPC on *h*-DMPC or on *d*-DMPC is the lower overall tilt angle, which may be forced by the tilt angle of the electrode-facing leaflet—the tilt angle in DMPE bilayers^71,76^ is lower than that in DMPC bilayers.^27,70^ The lipids in the electrode-facing leaflet exhibit similar trends to each other in their dependence upon potential, with the tilt angle increasing to a maximum at approximately −0.5 V to −0.6 V and then decreasing to lower values in the negative potential range.

**Figure 5:**
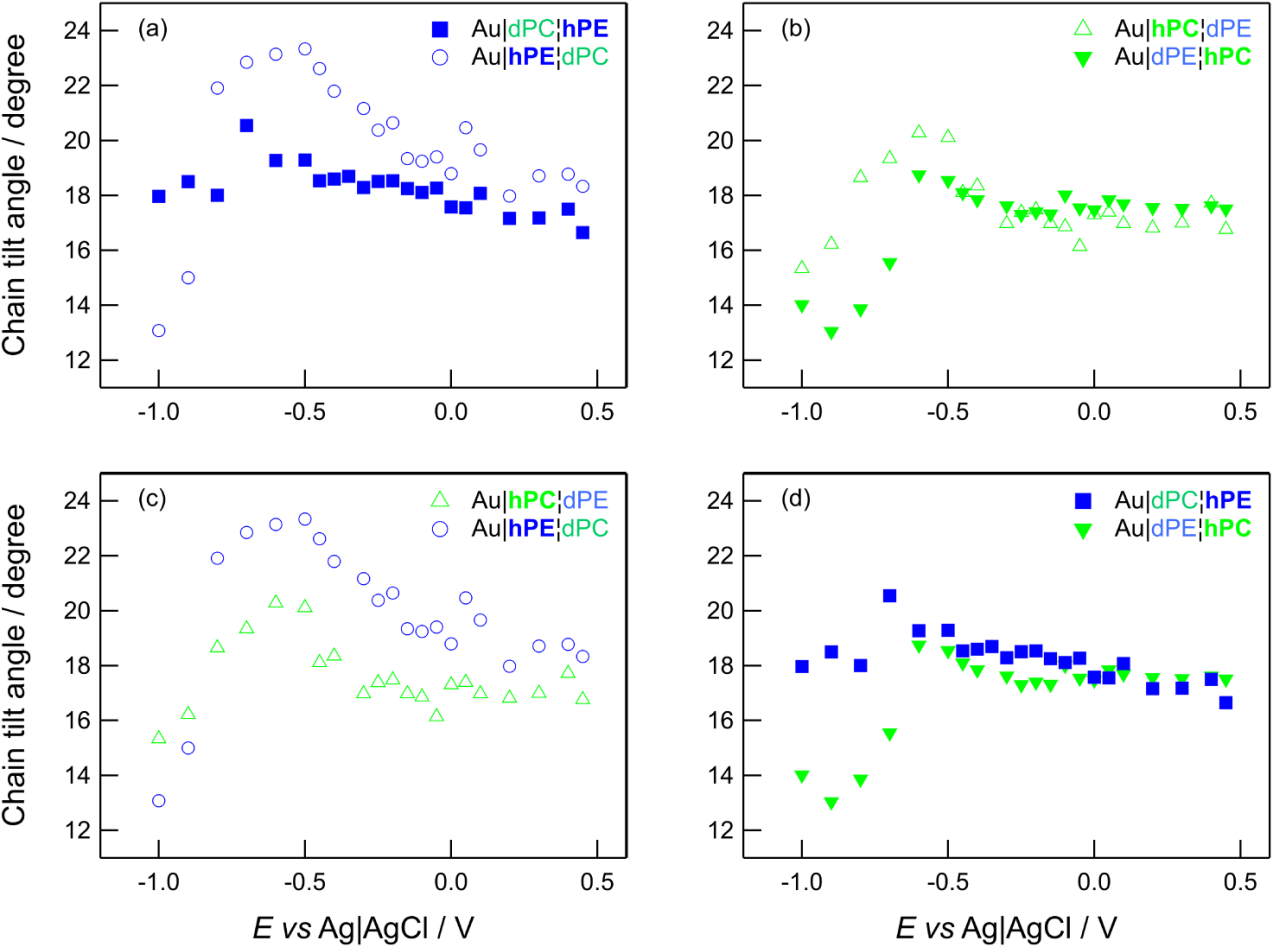
Comparisons of the dependence of chain tilt angle on applied potential. (a) compares how DMPE behaves in each half of the bilayer. (b) compares how DMPC behaves in each half of the bilayer. (c) compares the behavior of different lipids in the electrode-facing monolayer. (d) compares the behavior of different lipids in the electrolyte-facing monolayer. The error bars have been omitted for clarity but are on the order of 4°.

## 4 Discussion

### 4.1 Lipid Ordering

The peak positions in Table 1 for DMPE asymmetric and symmetric stretching modes are slightly higher than for DMPE bilayers on Au(111)^71,76^ but are still lower than for DMPC bilayers on Au(111).^27,70^ They are consistent with a small number of *gauche* conformers and a gel-like state for DMPE.^77,86^ The DMPC symmetric stretching vibration frequencies are also intermediate between DMPC bilayers and DMPE bilayers but the asymmetric stretching frequencies are similar to those in DMPC bilayers. There is little difference between DMPE and DMPC symmetric stretching vibrations in the asymmetric bilayers compared with the spectral resolution of 2 cm^–1^. Unfortunately, increasing the spectral resolution decreases the signal-to-noise ratio proportionately^94^ and the time required to acquire spectra of lipid bilayer samples at higher resolution becomes impractical.^24^ For this reason, most supported lipid bilayer measurements are not made at resolutions greater than 2 cm^–1^. Despite this, the peak positions can be used to compare different lipids. Lipids with high phase transition temperatures give spectra with peaks of lower wavenumber than those with lower phase transition temperatures. For example, Au-supported dipalmitoylphosphatidylcholine (DPPC), which has saturated and longer tails than DMPC, also gives peak positions at lower wavenumber (2851.3 and 2919.8 cm^–1^)^95^ and the unsaturated lipid dioleoylphosphatidyl choline, DOPC, gives peaks centered at higher wavenumber (2927.6 and 2854.7 cm^–1^)^78^ than DMPC.^27,70^ The spectra of DMPC bilayers formed via vesicle fusion have also been measured as a function of temperature and showed a clear increase of around 2 cm^–1^ for the symmetric vibration and 4 cm^–1^ for the asymmetric vibration as the temperature was increased through the phase transition.^24^ Hence, although the differences between asymmetric and symmetric bilayers are small, they can be usefully compared. To verify the differences between different bilayers, two or three replicate measurements have been made for each bilayer type and the values reported in Table 1 represent the mean values of those measurements.

The full widths at half-maximum (FWHM) are also similar between bilayers and, given the potential for error in fitting, any difference between DMPC and DMPE in symmetric or asymmetric bilayers is small. Symmetric undeuterated DMPC bandwidths increase a little at negative potentials,^27^ while the DMPC bandwidths in asymmetric bilayers decrease slightly at negative potentials. DMPE bandwidths in asymmetric bilayers show little change.

The band positions and FWHM suggest that a *d*-DMPE monolayer exerts a small increase in ordering in the opposing *h*-DMPC monolayer and a *d*-DMPC monolayer has a small disordering effect on *h*-DMPE compared with that of *d*-DMPE.

### 4.2 Initial Bilayer Structure

The values of chain tilt angle at low charge densities, around the potential of zero charge (pzc), can be used to compare the structures of the lipid bilayers both with their symmetric counterparts and with other reported bilayers characterized in the absence of an applied electric field. The pzc of the Au(111) electrode coated in Au|PE|PC or Au|PC|PE is ∼0.2 V.^73^ The tilt angles for all the asymmetric bilayers in this potential range are similar to one another, between 17–19° (with an estimated error of around 4°). In this potential range, Au|hPE|hPE bilayers on Au(111) also have chain tilt angles of ∼ 17°,^76^ while Au|hPC|hPC bilayers on Au(111) have chain tilt angles of ∼24°.^27^ Both halves of the bilayer contribute to these reported tilt angles. The tilt angles of each half of the chemically symmetric bilayers have also been determined independently by selectively deuterating each monolayer in turn. For DMPE, the average tilt angles for the proximal (electrode-facing) and distal (electrolyte-facing) leaflets are 15° and 20°, respectively.^71^ For DMPC, the corresponding average values are 25° and 35°.^70^ The DMPE tails in the PC/PE asymmetric bilayers are thus similarly tilted to DMPE in DMPE bilayers on Au, while the DMPC tails are less tilted than in DMPC bilayers on Au. The relatively low DMPC chain tilt angle is probably a result of interaction with the DMPE monolayer.

Table 2 summarizes tilt angles measured for multilayers, bilayers, and monolayers of some saturated PC and PE lipids reported in the literature. PE lipids tend to have untilted chains (except for Au-supported layers) in the gel phase^96,97^ or monolayer solid phase^98–100^ and tilt angles of up to ∼19° in the monolayer liquid condensed phase.^100^ PC samples below the main phase transition^101–104^ and monolayers in the liquid condensed phase,^17,105^ generally exhibit tilt angles of around 30°, which enables the chains to increase the strength of their dispersion interactions (by reducing their cross-sectional area to 40–42 Å^2^). DMPC can also be tilted at lower angles over short distances: in the ripple phase, the tails are tilted at 18° to the bilayer normal within the major (longer) arm of the sawtooth structure and the two monolayers are coupled.^106^ In the shorter arm, the lipids are likely to be disordered and are not in registry.^106^ DMPC on Au can also reorganize into a structure of lower footprint, with staggered headgroups and lower chain tilt angle.^27^

**Table 2:** Literature Values of Chain Tilt Angles in Gel and Ripple Phases for PC and PE Lipid Multilayers, and in Liquid Condensed or Solid Phases for Monolayers and Bilayers^a^

Lipid	Sample type	Phase	Chain tilt angle/degree	Molecular area/Å^2^	Comment
DMPC^101^	Suspended oriented multilayers	Various	26–30		Three phases within gel phase
DMPC^102^	Oriented multilayers	Gel (10 °C)	31.3–32.3	45.9–47.5	Matches extrapolation from longer chain PC^104^
DMPC^106^	Oriented multilayers	Ripple	18 (long arm)		Shorter arm disordered
DMPC^27,70^	Bilayer (Au)^27^	rRipple	24	45	At low charge density
	(partial deut)^70^		25 prox, 35 dist		
DPPC^104^	Multilayers	Gel	30–35	45–48	
DPPC,^17,105^	Monolayer	*L*_c_ 50–20 mN m^–1^	22.5–26.8	45.4–48.0	
DPPC,^17,105^	Bilayer (quartz)	Transf. at 45 mN m^–1^	26.8,^17^ 26.4 ^105^	45.9,^17^ 46.8 ^105^	Layers coupled
DPPC^95^	Bilayer (Au)	Transf. at 40 mN m^–1^	24	45	At low charge density
DMPE^98–100^	Monolayer	Solid	Untilted^98,99^	40.6 ^100^	*d*-DMPE also untilted at 40 mN m^–1^.^100^
		47 mN m^–1^	Untilted^100^		
		40 mN m^–1^	Untilted^100^	40.8 ^100^	
DMPE^71,76^	Bilayer (Au)^76^	Solid	17		
	(partial deut)^71^		15 prox, 20 dist		
PE^97^ (various)	Multilayers	Gel	Untilted	41	Independent of chain length
DLPE^96^	Multilayers	Gel	Untilted		Metastable phase
DPPE^107^	Monolayer	Solid, 45 mN m^–1^	Untilted	40.0	
DPPE^107^	Bilayer (quartz)	Transf. at 45 mN m^–1^	∼3	40.6	Layers not coupled, lower ordering than monolayer

a Values obtained with PM-IRRAS for bilayers on Au, X-ray diffraction or grazing incidence X-ray diffraction for all other samples

Hence, it is possible for the DMPC monolayer to pack in a way that is compatible with interacting with the opposing DMPE monolayer. DMPE, on the other hand, is unlikely to be able to match the preferred tilt angles of DMPC. The shape of the DMPE molecule is roughly cylindrical, as its critical packing parameter is close to 1, so tilting of the hydrocarbon chains is likely to result in breaking of interheadgroup hydrogen bonds, which is likely to be energetically unfavorable. DMPC as the more flexible monolayer, with weaker interheadgroup interactions, is better able to adapt to DMPE. The DMPC headgroups are larger than the cross-sectional area of the tails so in order to match the DMPE tilt angle, DMPC will need to rearrange with staggered headgroups, tilted headgroups, or introduce defects to relieve the mismatch. A larger number of defects in the proximal leaflet may lead to the slightly higher capacitance reported for Au|PC|PE compared with Au|PE|PC.^73^

The matching of DMPE and DMPC monolayers is a surprising result given that the two halves of DMPC bilayers have been reported to have different tilt angles. The difference in shape between DMPE and DMPC is much greater than between *h*-DMPC and *d*-DMPC. However, similarity in shape does not necessarily result in structural coupling of the monolayers. Grazing incidence X-ray diffraction (GIXD) studies of DPPE bilayers supported on quartz indicate that the two halves of the bilayer scatter as independent entities: the FWHM of the out-of-plane Bragg rods are consistent with a coherence length similar to that of the corresponding monolayer on water, rather than twice that length.^107^ Detailed fitting of the intensity-*q*_*z*_ profiles (Bragg rods) confirmed this interpretation. X-ray reflectivity (XRR) measurements of the DPPE samples also indicated some differences in scattering length density of the two headgroup layers, with the water-facing headgroup layer containing more water than the quartz-facing headgroup layer.^107^ The suggested reason for the independent structures of the two monolayers was that the support impeded registry between the leaflets. In-plane peak widths and positions also showed the bilayers were less ordered and slightly more loosely packed (∼1.3% expansion) than the monolayers.^107^ These subtle changes could also be explained by support effects. On the other hand, similar studies of DPPC bilayers supported on quartz have shown that the two halves of the DPPC bilayer do scatter as one entity, with coherence lengths across the bilayers twice those of the corresponding monolayers on water or on quartz.^17,105^ The DPPC molecules in the supported bilayer have smaller molecular areas (ca. 1%) but larger tilt angles than in monolayers, indicating that the chain cross-sectional areas are smaller in bilayers.^105^ A reduction in freedom of the hydrocarbon chains to rotate around their axes was also deduced from fitting of the Bragg rod profiles of DPPC bilayer samples, whereas monolayer samples’ data could be fitted to a model where the chains were free to rotate.^105^ This restriction in rotation may be related to the compaction of the chains and/or the structural coupling between the two halves of the bilayer.^105^

The reasons for the differences in interleaflet interactions within DPPE and DPPC bilayers are not specifically addressed, although the earlier DPPE study^107^ notes reports of bilayer-spanning domains in PE/PC giant unilamellar vesicles^108,109^ and the DPPC study discusses in some detail the thermodynamic considerations for coupling between the two monolayers, suggesting the main driving force to be the interaction energy between terminal methyl groups in opposing layers.^105^ The existence of coupling in PE/PC vesicles suggests PE lipids are not intrinsically unable to couple across a bilayer. One possible reason is an interaction of DPPE with the support with little water present in the proximal layer, whereas the support-facing DPPC layer is more hydrated than the outer layer and supported on a thin (4.2 Å) water cushion. It should also be noted that the DPPC layers were deposited from a less closely packed liquid-condensed phase, whereas the DPPE layers were deposited from the solid phase. For DMPE/DMPC systems studied on gold, lipid-support interactions during transfer may also play a role, although the interaction with gold is different from with quartz, as the hydrophilicities differ and it is possible that phosphate groups interact with gold. However, DMPC headgroups^27^ are more solvated than DMPE headgroups^76^ and neutron studies of DMPC-containing^26^ and DMPE-containing bilayers^110^ suggest more water in the headgroup slabs of DMPC than DMPE, similarly to the findings on quartz. The balance between natural lipid–lipid interactions and lipid-support interactions results in a lower tilt angle of the proximal layer of DMPC on gold than on water^70^ and a higher tilt angle of DMPE on gold than on water at the deposition pressure.^71,100^ The tilting of the DMPE tails induced by deposition may afford the flexibility needed for DMPE to interact with opposing layers.

A GIXD experiment could provide useful information on whether the tails in opposing leaflets are in registry; unfortunately, this is not possible on the Au substrate because the Au would dominate the scattering. Consequently, we cannot be certain whether the similarity in tilt angles and ordering of tails in our asymmetric DMPE/DMPC systems mean that the layers are coupled, although it seems likely. However, if the initial bilayer is formed of monolayers coupled with one another, the state is metastable rather than at equilibrium, similarly to DPPC bilayers on quartz.^105^ The metastability of the bilayer structure means it can be disturbed upon perturbation, for example by application of an electrical potential difference across the bilayer (see below).

One possibility that should not be discounted is that the bilayers could scramble, to form a mixed bilayer containing both molecules on each side. However, this is less likely than the mixing of deuterated and undeuterated forms of the same molecule, and the temperature used for deposition and measurement should significantly slow the kinetics of scrambling. As both *d*-DMPE and *h*-DMPE were in the solid phase for deposition, any scrambling would involve breaking of intermolecular hydrogen bonds as well as the activation energy associated with “flipping” to the opposite side. The four asymmetric bilayers exhibit distinct dependences on applied potential, which suggests bilayer asymmetry has been maintained on deposition, otherwise the trends should be the same.

### 4.3 Field-Induced Changes in Bilayer Structure

Figure 5 shows that the chain tilt angle of each lipid changes as the potential is made more negative. At more negative potentials, the charge density on the surface and the resulting electric field across the bilayer increase in magnitude, which causes reorganization of charges, dipoles and polarizable species or functional groups. The field induced across the bilayer is comparable with that induced by *trans*-membrane potentials across natural membranes^25,26^ and the rational potential (the potential minus the pzc) is a good approximation to the *trans*-membrane potential.^75^ Hence, the changes observed for supported bilayers with applied potential give an indication of the effect of membrane polarization on membrane structure.

Lipids supported on Au(111) have been reported to undergo a phase transition between around −0.4 V and −0.6 V;^25^ this phase transition is a common feature in differential capacitance and chronocoulometry data for lipids with different headgroups,^27,75,76,100,111^ different tail lengths and saturation,^27,75,78,95^ different backbones^74,112^ and different mixtures.^72,74,75,100,113,114^ This phase transition is also observed in chemically asymmetric bilayers and is illustrated in Figure 6, which overlays the differential capacitance curves for Au|PE|PC and Au|PC|PE bilayers reported in ref 73 with the corresponding tilt angle dependences. These curves were recorded in the negative-going scan and show a rise in capacitance at −0.4 V, followed by a peak at −0.65 V to −0.7 V.

**Figure 6:**
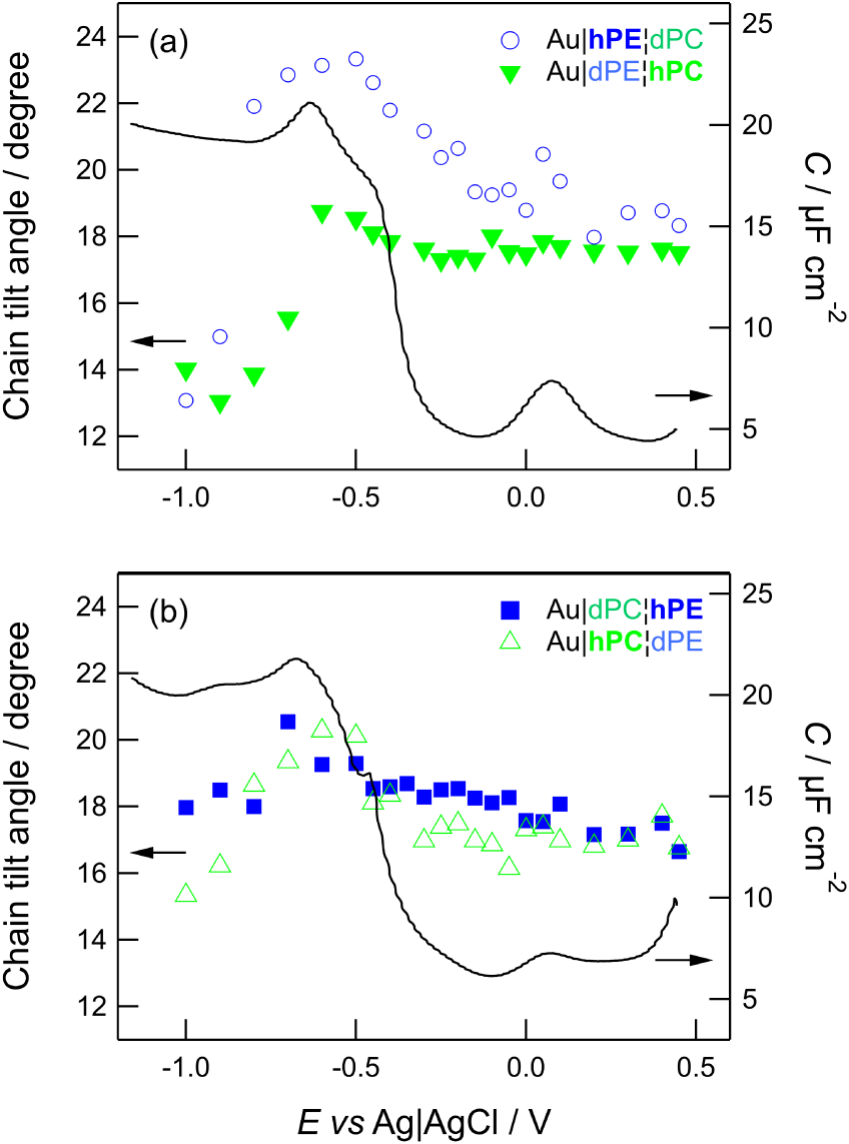
Dependence on tilt angle overlaid with differential capacitance for (a) Au|PE|PC and (b) Au|PC|PE. Blue circles and squares represent PE, green triangles represent PC. Open shapes represent electrode-facing leaflet, filled shapes represent electrolyte-facing leaflet. Capacitance data reproduced from the authors” data reported in Madrid and Horswell. Copyright © Elsevier 2017.^73^

Results from a neutron reflectivity study of bilayers formed from rupture of DMPC/cholesterol vesicles onto Au suggested that the phase transition is related to incorporation of water into the bilayer. As the potential was made further negative, this water moved out of the bilayer to form a layer between the bilayer and the surface (detachment of the bilayer).^26^ These changes in water distribution have also been observed with Surface-Enhanced Infrared Absorption Spectroscopy (SEIRAS).^115^ At positive potentials, the water is mainly associated with lipid headgroups and some isolated water molecules are observed during the main phase transition. At negative potentials, changes in the spectra show the formation of a liquid-like water layer between the support and the bilayer.^115^ While a thin water layer has been observed for DPPC-based bilayers on quartz,^17^ the presence or absence of a water layer between substrate and PE-based bilayers could not be confirmed on quartz^107^ or on gold.^110^ However, the electrochemical phase behavior of PE-containing bilayers is similar to that of other lipid types and the two processes seen in the curves in Figure 6 indicate a distinct phase at negative potentials,^73^ so we infer that the behavior of the asymmetric bilayers can be interpreted in the same way.

Despite similarity in the electrochemical data of Au|PE|PC and Au|PC|PE, the field-induced structural changes in each type of bilayer differ. The common feature is a tendency for chain tilt angle of the inner monolayer to increase with increasing charge density, starting from around 0 to −5 μC cm^–2^ (Figure S5). This onset is likely to be related to the interaction of headgroup dipoles in the proximal leaflet with the increasingly negatively charged surface: both DMPC^27^ and DMPE^76^ bilayers have been reported to show a small change in phosphate group orientation. DMPE layers are more ordered than DMPC and their molecules interact via direct hydrogen bonds, so would be expected to have a larger resultant dipole moment that might in turn lead to a stronger response manifested in a greater change in orientation of headgroups and (consequently) tails. However, additional work would be required to break the interlipid hydrogen bonds in the PE layer, as previously discussed for PE bilayers,^76^ which might offset any larger driving force. The extent of the change in chain tilt angle (from initial state to peak) is rather similar for the Au|**hPE**|dPC and Au|**hPC**|dPE layers, differing by around 2°, which suggests the difference in the headgroup orientational changes are small. The overall dipole moments across the bilayers are small, as determined from charge density measurements,^27,73,76^ so changes in the orientations of headgroup vibrations (including carbonyl groups, which contribute to the dipole moment) may be taken as approximately similar on each side. Both (undeuterated) lipid bilayers have similar phosphate group orientations (O–P–O symmetric stretch ca. 70–75° from the surface normal) and the orientational changes are small, a few degrees,^27,76^ although a little stronger in deuterated DMPE (which may be more flexible) than undeuterated DMPE.^76^ The dependence of carbonyl group dipole orientations on potential mirror those of the symmetric methylene stretching modes. The reported electrochemistry data can also provide bilayer surface pressure and it is useful to note that decreasing surface pressure results in an increase in tail tilt angle (Figure S8), which mirrors the behavior of monolayers at the air|water interface.^100^

Figure 6 shows that the tilt angle for the inner monolayers rises during the main phase transition and its maximum coincides with the peak in capacitance that corresponds to detachment of the bilayer.^73^ The peak in the IR tilt angle thus likely results from the disruption on incorporation of water and subsequent egress of water from the bilayer to form a water film, leaving behind a slightly thicker and less tilted bilayer at the most negative potentials. The behaviors of the outer monolayers are reminiscent of those of the corresponding symmetric bilayers, with DMPE showing a generally featureless slow increase in tilt angle and DMPC showing a small increase in tilt angle followed by a marked drop. The most likely explanation is that the distal leaflet behaves independently of the proximal leaflet and is primarily influenced by the electrolyte rather than the surface. Symmetric systems have also shown some differences between support-facing and solution-facing sides of a bilayer. For example, the solvent content in the two leaflets of DMPE/DMPS mixed bilayers differs and the field-induced changes in solvation are more marked in the electrode-facing monolayer.^110^ This finding could explain the stronger dependences of the tilt angle on potential or charge for the electrode-facing monolayers in the asymmetric bilayers.

Subtle differences in monolayer structure within bilayers on quartz substrates can also be observed as discussed above. DPPE bilayers exhibit small differences between the two halves in lipid packing, which were suggested to reflect differences in hydration of the headgroups on the support or on the solution side of the bilayer.^107^ Analysis of the diffraction Bragg rods showed the two monolayers were independent.^107^ On the other hand, similar X-ray measurements with supported DPPC bilayers indicated domains spanning the bilayer, indicating coupling of the monolayers.^17,105^ This may be because the headgroup environments are more similar than for DPPE, as a thin water cushion was also observed between substrate and bilayer.^17^ However, these DPPC bilayers were described as being a quenched state rather than an equilibrium state, based on comparison with the structures of the monolayers from which they were formed. Our DMPE/DMPC bilayers may also be formed in a quenched state, since the DMPC in particular appears forced into a structure that differs from the expected equilibrium structure. Although the similarity in chain tilt angles of the DMPE/DMPC bilayers at low charges could suggest the two monolayers may be structurally coupled, it is not possible to say with certainty from this result alone. The small differences in wavenumber also indicate some small difference in packing density. The results in Figure 6 suggest that if the DMPE and DMPC layers are initially coupled, they are likely to be in a metastable state that is easily disturbed by the imposition of the electric field and/or the movement of water through the film that the field causes. That is, the electric field and/or the interaction of water with the field disrupt the structure and lead to decoupling of the monolayers during the phase transition.

In other systems, a study of PC bilayers on mica reported asymmetric behavior of the two leaflets during the chain-melting transition, where support or water interactions with the proximal leaflet were suggested to result in decoupling of the bilayers as the temperature increased through the phase transition range.^14^ This led to the independent melting of the two halves of the bilayer.^14^ In asymmetric lipid systems, ordered regions have been shown to induce ordered regions in the other half of the bilayer but their tendency to do this depends on a range of factors, including temperature, ability of chains to interdigitate and ability of the opposing lipids to form ordered phases (which depends on their chain-melting temperature).^58–60,65,66,68^ The independence of the DMPE and DMPC monolayer responses in the present work may be a result of the mismatch in their fluidity (a more stark example of this would be the comparison of asymmetric DOPC/sphingomyelin and symmetric DOPC vesicles reported by Chianta and London^59^) or greater differential stress. For our systems, this mismatch would be further increased above the DMPC chain-melting phase transition, so the responses are likely to be similar at higher temperatures if the lipids do not scramble and bilayer asymmetry is maintained. An early study of DMPC bilayers on Au formed via vesicle fusion showed similar potential-dependent behavior at 20 and 30 °C and also demonstrated the chain-melting phase transition takes place on the support at around 25 °C.

Considering the results reported in the literature for a variety of systems and conditions, it appears that PC lipids and PE lipids may or may not interact with lipids in the opposing layer, depending on the intermonolayer interactions, lipid-support/water interactions, temperature, and fluidity. There does not appear to be an unambiguous definition of coupling, with diffraction studies considering it in terms of the bilayer (or domains in the bilayer) scattering as one unit or two independent units, while other studies discuss the extent of ordering in opposing leaflets, which could also be taken as an indicator of interleaflet interaction. These factors can be related, in that a structurally coupled bilayer (which we here assume to be a bilayer scattering as one unit) will normally have similar tilt angle and ordering in each leaflet. However, a structurally uncoupled bilayer (e.g., DPPE on quartz) can also have similar tilt angle and ordering, and therefore properties, as the two monolayers may exhibit only very subtle differences. It is not possible to be certain whether our PC/PE asymmetric systems are structurally coupled but the similarity in peak width and subtle changes in wavenumber compared with symmetric bilayers suggests coupling may be possible in the sense of intermonolayer interactions and mutual influence on one another’s properties. It is likely that our PC/PE asymmetric systems as initially deposited are in a metastable state, possibly coupled, but that the intermonolayer interactions are weak enough to be disrupted by the stronger interactions of the lipids with an applied electric field. The fact that two monolayers can become decoupled to melt independently^14^ suggests that it should also be possible for lipid layers facing support or electrolyte to respond differentially to an applied electrical potential gradient. It is possible that in melting transitions or electrochemical phase transitions, the two monolayers regain their mutual influence on completion of the transition, if they reach a similar state (e.g., the L_α_ phase for a chain melting transition or the detached state for the electrochemical transition). The changes in ordering between adsorbed and detached states are minimal, so this different response does not necessarily mean mutual influence is irreversibly lost, but it is is not possible to be certain whether the bilayers on the water cushion regain their former level of interleaflet interaction. However, comparing the electrochemical response of each leaflet does appear to be a useful indicator of the strength of interaction between the monolayers in their initial state.

### 4.4 Implications for Symmetric Systems

The chemically asymmetric bilayers respond to the increasing charge density differently from chemically symmetric but isotopically asymmetric bilayers. Comparing these systems with fully symmetric systems suggests that the outer monolayer determines the overall behavior of bilayers in which the two halves are probably coupled, rather than the first monolayer driving changes in the second. We first consider the behavior of DMPE in the different types of bilayer. Figure 7 shows the chain tilt angle as a function of surface charge density for the chemically asymmetric bilayers along with the data for isotopically asymmetric and symmetric DMPE bilayers from refs 71 and 76.

**Figure 7:**
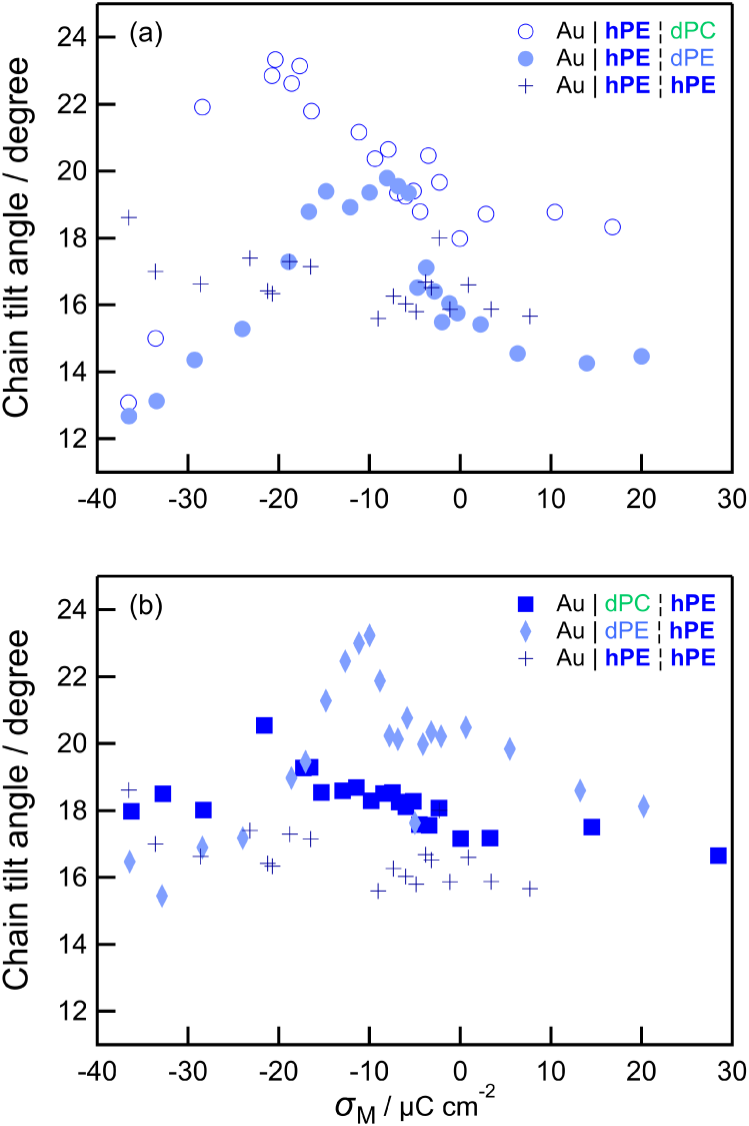
Comparison of dependence of tilt angle for *h*-DMPE within different types of bilayer. (a) compares the electrode-facing leaflet and (b) compares the electrolyte-facing leaflet. Crosses represent symmetric *h*-DMPE bilayers. Pale blue filled shapes represent *h*-DMPE in chemically symmetric but isotopically asymmetric bilayers (circles Au|**hPE**|dPE and diamonds Au|dPE|**hPE**)., Open blue circles represent Au|**hPE**|dPC and filled blue squares represent Au|dPC|**hPE**. Data for DMPE bilayers adapted with permission from those reported in ref 71 (Copyright © 2015 American Chemical Society) and ref 76 (Copyright © 2013, the authors under a Creative Commons (CC-BY) license). Charge density values for asymmetric bilayers obtained from the authors” data reported in Madrid and Horswell. Copyright © Elsevier 2017.^73^

The isotopically asymmetric bilayers (Au|dPE|**hPE** and Au|**hPE**|dPE) have slightly different tilt angles in each leaflet but similar behavior to each other, with a small increase in tilt angle between 0 and −10 μC cm^–2^ (roughly the onset of the first, main phase transition corresponding to the rise in capacitance in Figure 6). This peak is likely to result from some electrostriction increasing tilt angle (and thinning the film), followed by a second change as water distribution in the bilayer changes during the phase transition. The tilt angle then falls during the main phase transition between – 10 μC cm^–2^ and −20 μC cm^–2^ and falls further as the bilayer detaches.^71^ The difference between this plot and the featureless slow increase in tilt angle for symmetric Au|hPE|hPE was attributed to increased flexibility in the deuterated half of the bilayer, which in turn influences the opposing half of the bilayer. Hence, the bilayer probably remains coupled in that the behavior of one monolayer influences that of the other and the two layers behave co-operatively. The increased flexibility in the *d*-DMPE layer is not enough to decouple the two halves of the bilayer. (Note the methylene stretching band positions are similar in each case and there is little difference in packing density between *d*-DMPE and *h*-DMPE;^100^ the flexibility probably arises from small differences in dispersion interactions between the two lipids.) When a DMPE monolayer is deposited on a DMPC monolayer, although the two halves are closer in orientation and state (as determined by band center) than DMPE and DMPC symmetric bilayers are to one another, they do not behave co-operatively. This suggests that if they are initially structurally coupled they are not able to remain coupled as the field strength is increased, and they respond independently. The *h*-DMPE layer in Au|dPC|**hPE** behaves in the same way as *h*-DMPE in Au|**hPE**|**hPE** bilayers. In these isotopically symmetric bilayers, both *h*-DMPE monolayers are stiff and may be structurally coupled with an enhanced stiffness. It is likely the properties of the distal monolayer determine the ability of the bilayer to take in water, resulting in similar behavior to that of the distal monolayer; if the monolayers were decoupled or if the proximal monolayer determined the overall behavior, the result would be similar to that of Au|hPE|dPC.

Turning to DMPC, partially deuterated bilayers (Au|dPC|**hPC** and Au|**hPC**|dPC) have a significantly higher tilt angle in the distal leaflet. In both leaflets, the tilt angle starts to decrease from approximately −0.4 V vs SCE (−0.36 V vs Ag/AgCl).^70^ Symmetric DMPC bilayers (Au|**hPC**|**hPC**) show similar behavior, but with a small increase in tilt angle at similar potential to the Au|dPE|**hPC** bilayer.^27^ The behavior of DMPC in the proximal leaflet of Au|**hPC**|dPE asymmetric bilayers is different, with a clear peak in the tilt angle during the main phase transition. This comparison, where the DMPC has similar response to the potential gradient in both leaflets of a DMPC bilayer but different response in a chemically asymmetric bilayer, suggests that symmetric DMPC bilayers may be coupled, whereas the DMPC/DMPE asymmetric bilayers become decoupled as the electric field increases. This is likely to be because the fluidity and density (and possibly also water distribution) is better matched in symmetric DMPC bilayers, despite the different chain tilt angles of the two halves. The DMPC monolayers are closer to equilibrium when opposed by DMPC than when opposed by DMPE. In symmetric DMPC bilayers, the behavior is determined by the properties of the distal monolayer and/or coupling makes the bilayer stiffer and more resistant to solvent ingress, altering the ability of the proximal monolayer to reorient as the potential is varied. The strong influence of the electrolyte-facing monolayer might also explain the larger increases in tilt angle for Au|**hPE**|dPC than for Au|**hPC**|dPE: dPE presents a stronger barrier to solvent incorporation than dPC, reducing the intensity of the changes in tilt angle. Molecular dynamics studies of electroporation in POPE and POPC symmetric and asymmetric bilayers showed the PE layers to be more resistant to field-induced water ingress (which mirrors its lower tendency to hydrate in the absence of fields), as a result of the denser headgroup packing of PE.^116^ In asymmetric systems the pores started on the PC side of the bilayer.^116^ It is likely that DMPE is similarly more resistant to the incorporation of water than DMPC. This interpretation of our results suggests that the support-lipid interactions have more influence over asymmetric systems (as both inner layers exhibit similar behavior) than over symmetric systems (where the stronger interleaflet interactions result in co-operative behavior more like that of the outer layer). It is also possible there are more defects in the dPC monolayer than the dPE monolayer. Some insight into all of these effects may be possible with neutron reflectivity experiments.

Figure 8 summarizes the different responses of the lipid bilayers to the increasing electric field strength. The top two rows depict the changes in structure of the chemically asymmetric layers, Au|PE|PC and Au|PC|PE. In each case, the proximal leaflet undergoes a reorientation of the lipid tails as the charge density increases and capacitance rises, which is followed by a decrease in tilt angle as the bilayer detaches. The overall decrease in tilt angle from that at low charge densities is most likely to be a result of a change in the nature of lipid-support interactions when water forms a film below the lipid layer. The bottom two rows depict the changes in the DMPC and DMPE symmetric bilayers. Comparing the top and bottom rows shows that the proximal DMPE leaflets behave differently depending on the identity of the lipid in the distal leaflet. Comparing the middle two rows shows that the proximal DMPC leaflet behavior also depends on the lipid in the distal leaflet. As the field appears to decouple the two monolayers in chemically asymmetric systems, the proximal leaflet is unaffected by the distal leaflet and reorients in response to the charge on the metal. The structural changes in the distal leaflet are a response to the interaction with electrolyte upon increasing field strength. As the distal leaflets respond to the potential gradient similarly in symmetric and asymmetric bilayers, it is likely that their structural changes induce similar changes in the opposing leaflets in symmetric systems. This suggests the layers in symmetric systems interact more strongly with one another than in asymmetric systems, as they behave co-operatively, and the overall response depends on the properties of the distal monolayer as the electrolyte interacts with the field. This stronger coupling is likely to result from a better match in fluidity between the two halves of the bilayer and, for DMPC in particular, a smaller distortion from equilibrium bilayer structure in the symmetric layers. Thus, there is a balance between lipid-support and interleaflet interactions. Where the interleaflet interactions are weaker (e.g., in our chemically asymmetric layers), there is a stronger influence of the support on the behavior of the support-facing leaflet, possibly an interaction between the phosphate group and gold that changes when the gold is negatively charged. Where the interleaflet interactions are stronger (e.g., in symmetric systems), the influence of the support is weakened and the bilayer behavior depends on the properties of the electrolyte-facing leaflet and its resistance to solvent ingress.

**Figure 8:**
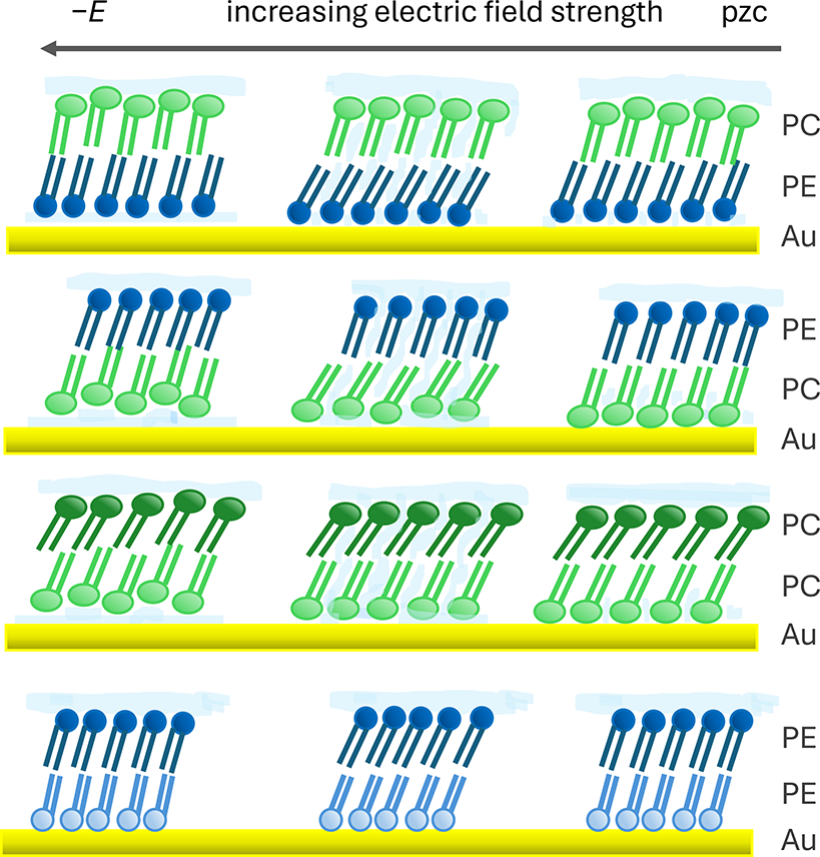
Schematic representation of the electrochemical response of different bilayers. Chemically asymmetric bilayers (top two rows) have similar tilt angle and ordering as deposited (near the potential of zero charge) but decouple and behave separately as the electric field strength increases. The symmetric bilayers (bottom two rows) behave co-operatively as the field strength increases, suggesting coupling across the bilayers. Note also the same trend in tilt angle is seen between rows 1 and 3 (PC outer layer) and 2 and 4 (PE outer layer).

These results show that studying asymmetric systems can yield useful insights into the behavior of apparently simpler symmetric systems. They also show that different environments on either side of a bilayer can have significant effects on lipid flexibility and orientation, which could be important in biological systems where local changes occur, e.g., ion gradients or binding of cations or peptides to anionic lipids on one side of a membrane. Finally, these results imply that an electric field might affect the interaction between the two halves of a bilayer, which could mean that coupling or decoupling in natural membranes can be influenced by membrane polarization. This could be important for the bilayer structure local to ion channels or lead to local changes in fluidity upon transfer of charged lipids. Further studies on the latter are in progress.

## 5 Conclusions

The effect of asymmetry in supported lipid bilayers on their electrochemical phase behavior has been studied. Asymmetric bilayers formed from DMPE and DMPC monolayers have similar organization and mobility that is intermediate between those of the symmetric (undeuterated) bilayers of each lipid: DMPC disorders DMPE and in turn DMPE imposes some ordering on DMPC. Hence, the proximal leaflet does not template the distal leaflet but rather the two leaflets moderate one another’s structure. These results illustrate the complexities involved in designing supported asymmetric bilayers. The differences between DMPC and DMPE are less stark than those between unsaturated and saturated lipids but the response of lipid tail orientation to an applied electric field demonstrates that if the lipids in these asymmetric bilayers are initially coupled across the bilayer, they do not remain coupled when the field strength is increased, despite moderating one another’s state. We propose that the initial state is a metastable one, and that the distortion induced by the applied field is sufficient to disrupt it.

DMPE and DMPC each behave differently depending on their location. The proximal leaflets respond more strongly to the surface charge density, with overall changes likely indicating different lipid-support interactions at negatively charged and uncharged surfaces, and the distal leaflets exhibit similar behaviors to the respective lipids in fully symmetric bilayers. Comparing the results with symmetric bilayers indicates that the symmetric bilayers are likely to be and remain coupled upon electrochemical perturbation, and that their electrochemical behavior is primarily determined by the properties of the electrolyte-facing monolayer. The distinct responses of the two halves of the bilayers observed in these experiments on asymmetric layers demonstrate a need to evaluate the consequences of coupling and decoupling in asymmetric bilayers intended for study of *trans*-membrane proteins in applied electric fields.

This approach to the study of asymmetric systems has also enabled a more detailed understanding of symmetric systems. The imposition of an electric field on a bilayer and study of its effect on bilayer structure enables us to extract more information than would structural experiments alone. Examination of the spectra at uncharged surfaces might have led to the conclusion that symmetric and asymmetric supported bilayers were similar in phase and probably coupled. Analysis of the changes in the spectra as the electric field was varied has been able to tell us that the asymmetric bilayers are or can become decoupled and has provided insight into how the two monolayers in symmetric systems influence overall behavior and the balance between support-lipid interactions and interleaflet interactions. Hence, the study of electrochemically induced phase changes has much to offer in developing our understanding of biomimetic membranes even where electrochemical processes may not be the primary focus. In a broader sense, investigating the perturbation of a biomimetic system also yields important insights and understanding.

## Supplementary Material



## Notes

Additional PM-IRRA spectra for each bilayer type and applied potential; Additional data analysis plots (tilt angles of transition dipole moments and chain direction vs potential, charge or surface pressure) (PDF)
