# Supplementary material for: A Spectroelectrochemical Study of the Effect of Asymmetry on the Electrochemical Response of Lipid Bilayers
Source: J Phys Chem B. 2026 Mar 10;130(11):3088–102. doi: 10.1021/acs.jpcb.5c05655 (PMC13007035; doi:10.1021/acs.jpcb.5c05655)
Supplement: Supplementary file 1 [file jp5c05655_si_001.pdf]

Supporting Information for A Spectroelectrochemical Study of the Effect of Asymmetry on the Electrochemical Response of Lipid Bilayers

Elena Madrid and Sarah L. Horswell*

School of Chemistry, University of Birmingham, Edgbaston, Birmingham, B15 2TT, UK

*s.l.horswell@bham.ac.uk

Addtional IR spectra

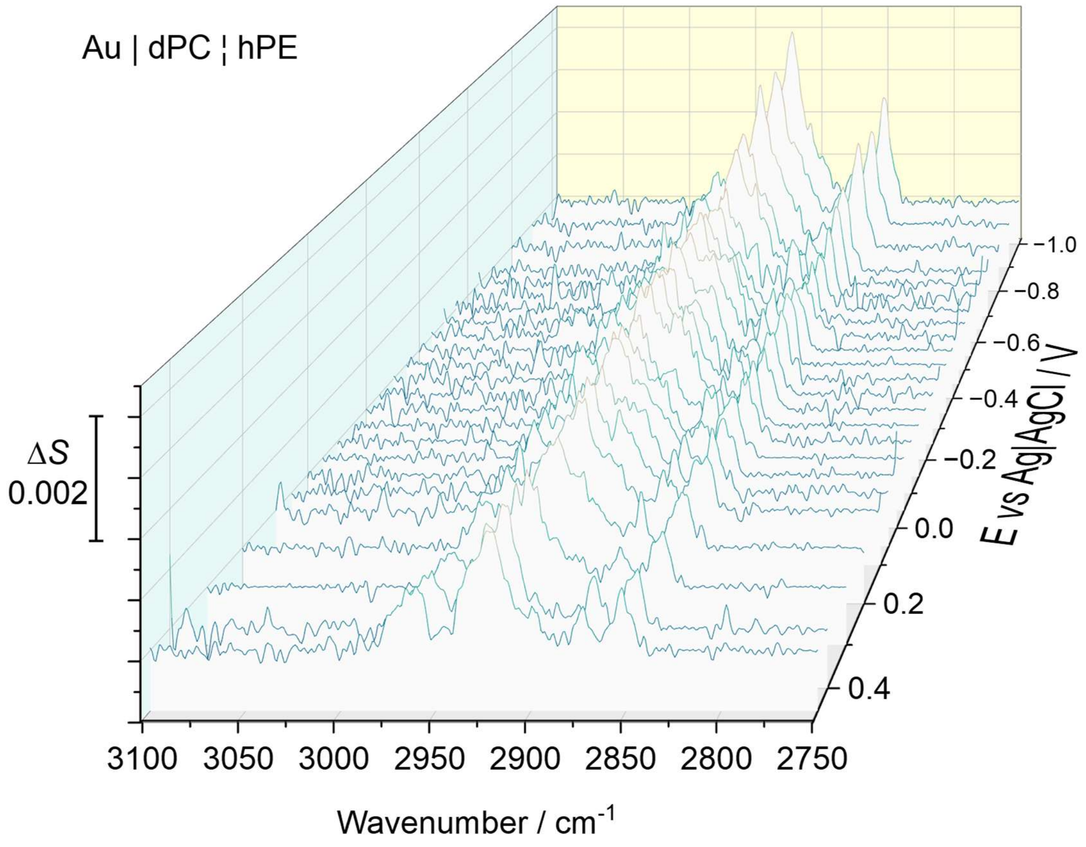

Figure S1 PM-IRRA spectra in the C–H stretching region for Au|dPC¦hPE in 0.1 M NaF in D_2O at different applied potentials. Window material BaF_2, angle of incidence 51°, electrolyte thickness 1.8 μm.

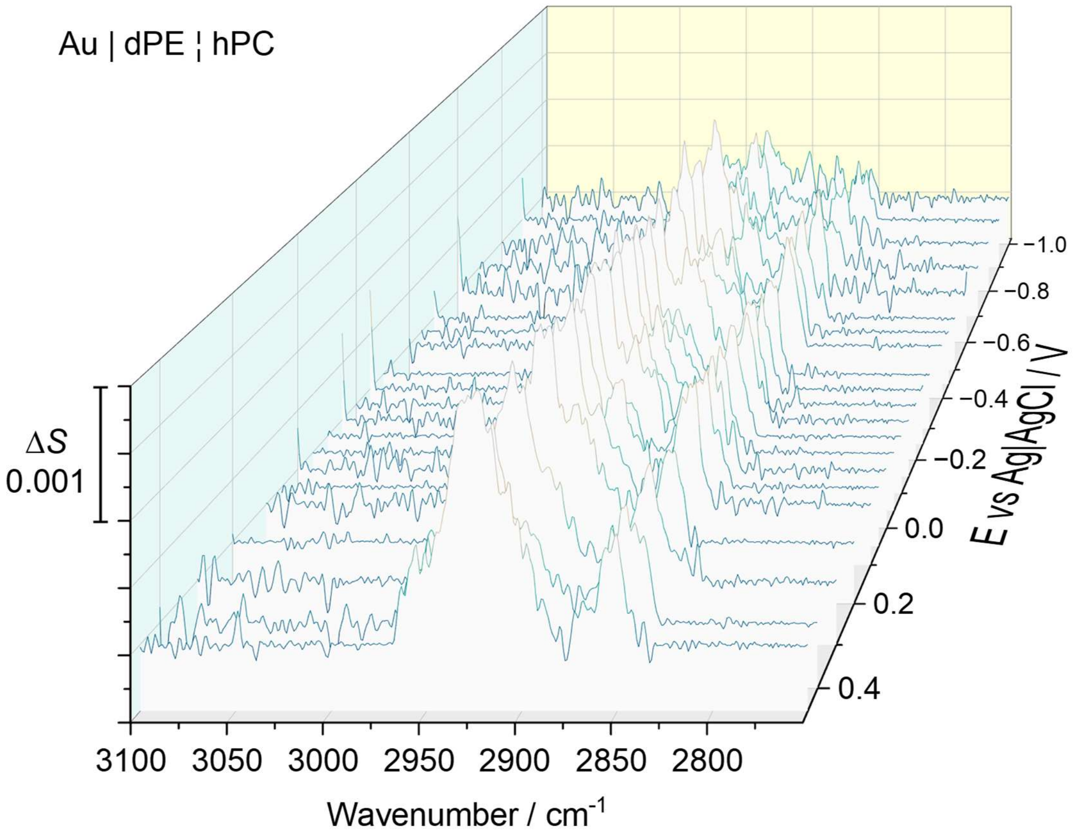

Figure S2 PM-IRRA spectra in the C–H stretching region for Au|dPE¦hPC in 0.1 M NaF in D_2O at different applied potentials. Window material BaF_2, angle of incidence 51°, electrolyte thickness 1.8 μm.

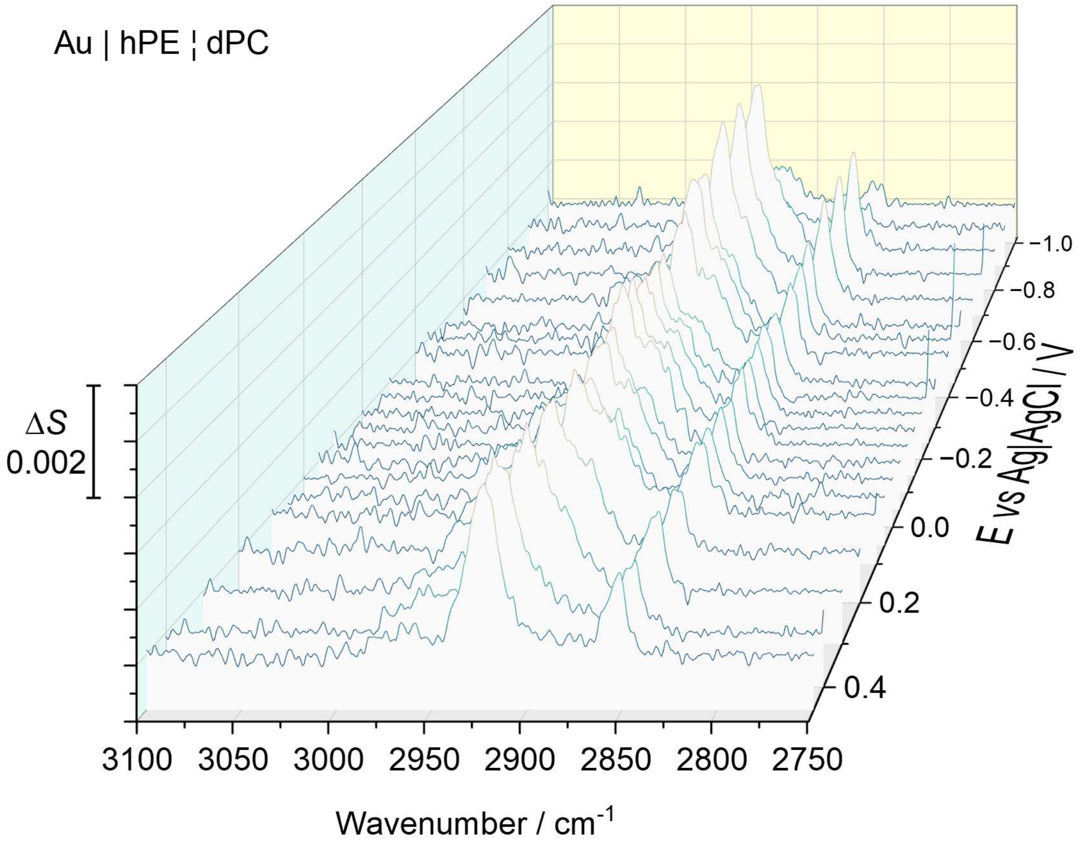

Figure S3 PM-IRRA spectra in the C–H stretching region for Au|hPE¦dPC in 0.1 M NaF in D_2O at different applied potentials. Window material BaF_2, angle of incidence 51°, electrolyte thickness 1.8 μm.

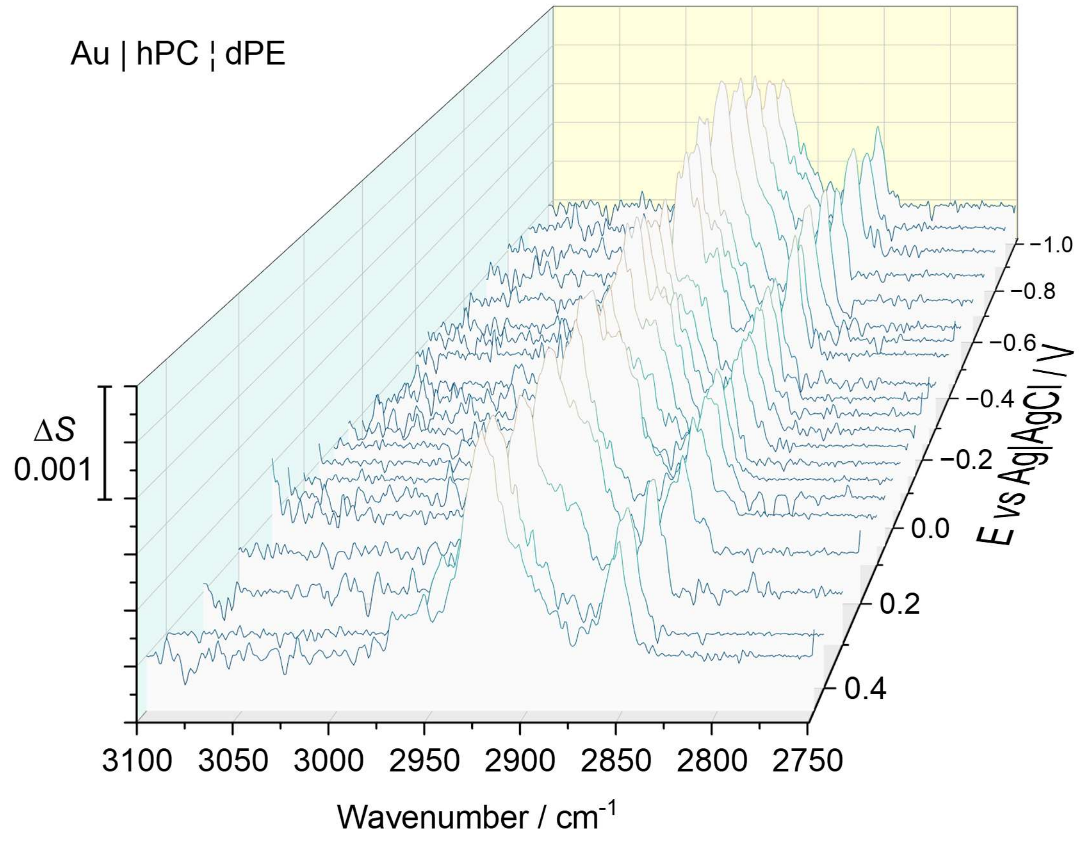

Figure S4 PM-IRRA spectra in the C–H stretching region for Au|hPC¦dPE in 0.1 M NaF in D_2O at different applied potentials. Window material BaF_2, angle of incidence 51°, electrolyte thickness 1.8 μm.

Additional data analysis plots

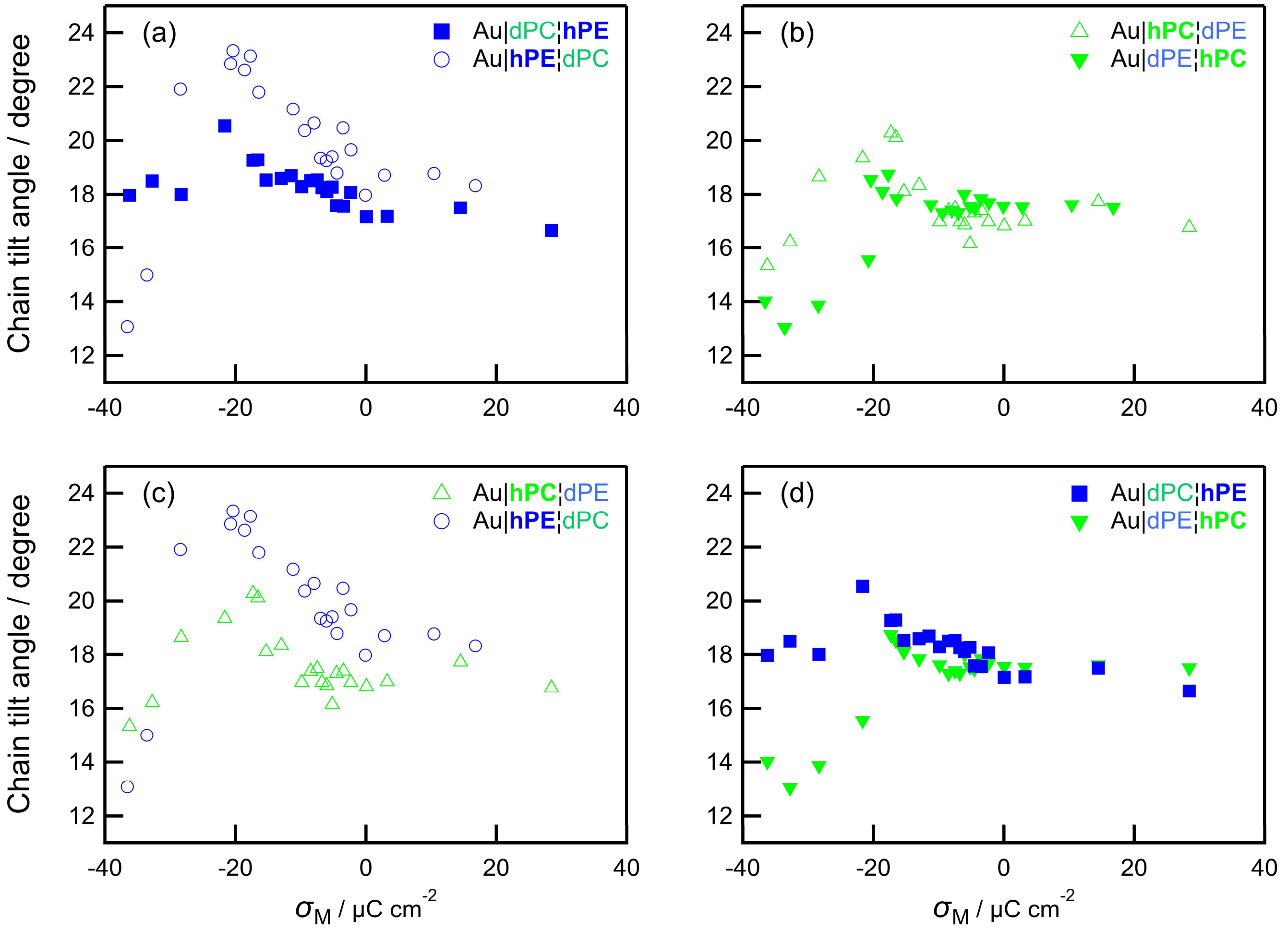

Figure S5 Comparisons of the dependence of chain tilt angles on surface charge density. (a) compares how DMPE behaves in each half of the bilayer. (b) compares how DMPC behaves in each half of the bilayer. (c) compares the behavior of different lipids in the electrode-facing monolayer. (d) compares the behavior of different lipids in the outer monolayer. The error bars have been omitted for clarity but are on the order of 4°. The values of charge density for each potential are taken from the authors' data reported in *J. Electroanal. Chem.* **2018**, *819*, 338–346. Copyright © Elsevier 2017).[S1].

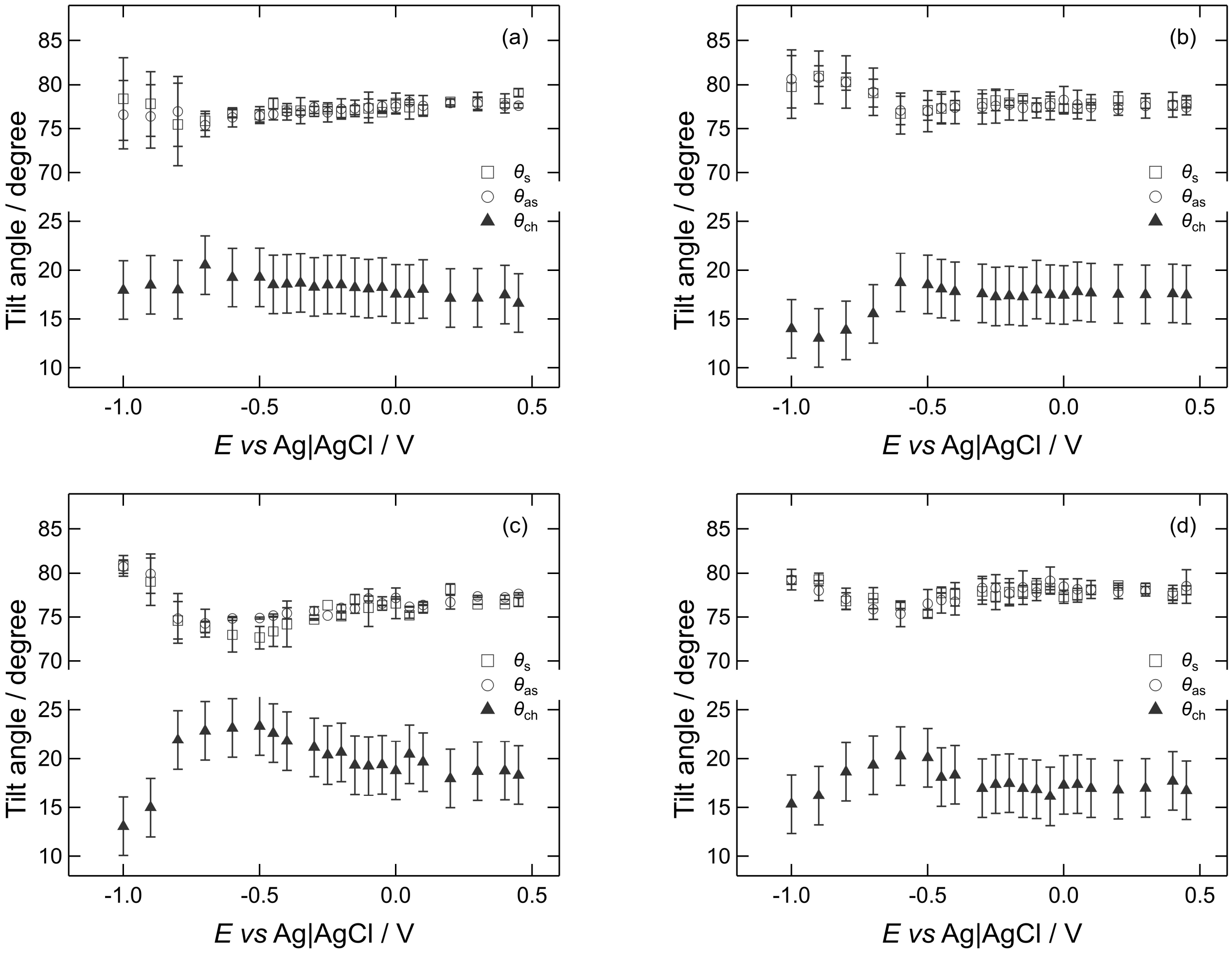

Figure S6 Dependence on applied potential of the tilt angles of the transition dipole moments and of the hydrocarbon chains. (a) Au|dPC¦hPE, (b) Au|dPE¦hPC, (c) Au|hPE¦dPC, (d) Au|hPC¦dPE. Open squares: symmetric stretching, open circles: asymmetric stretching, filled triangles: chain.

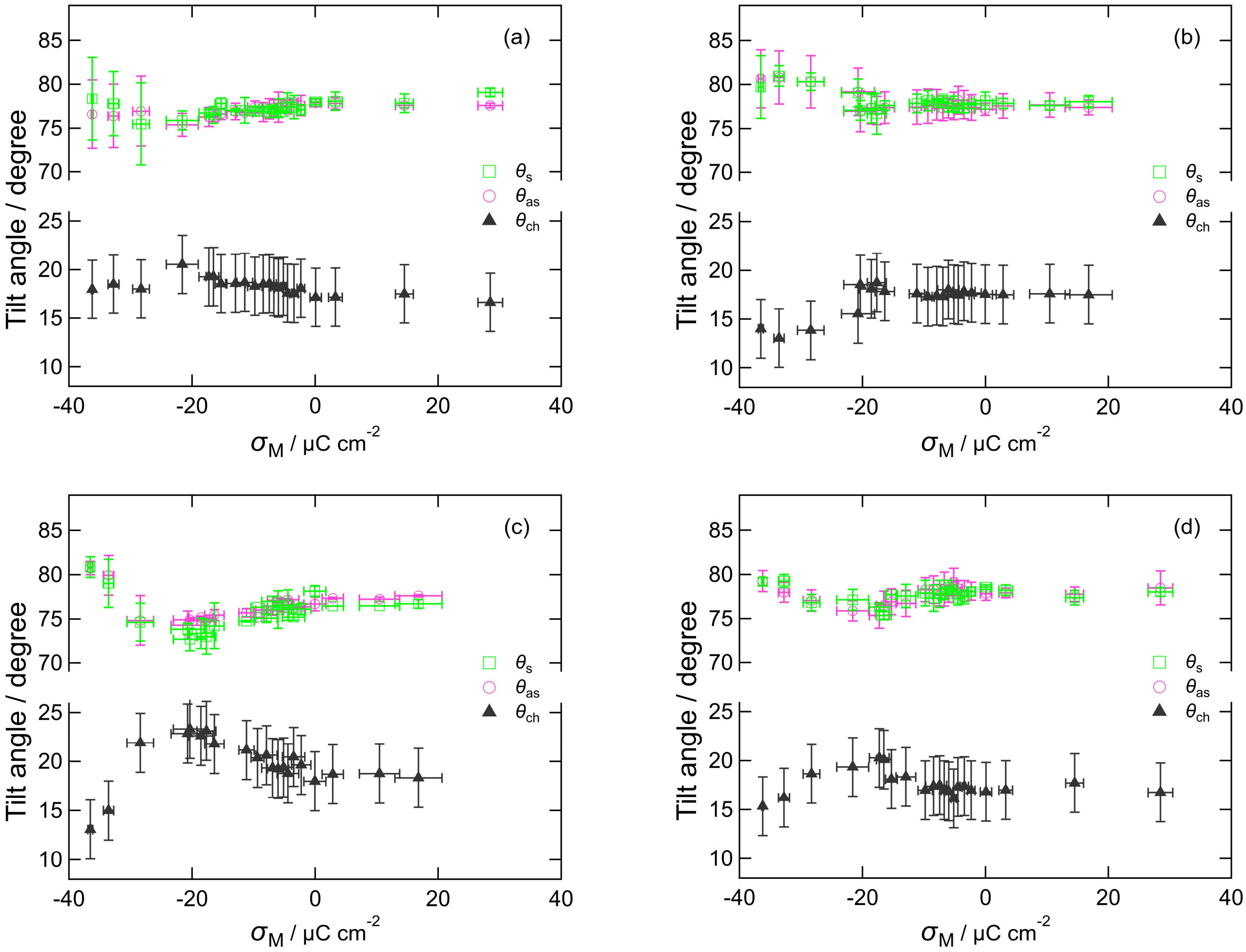

Figure S7 Dependence on charge density of the tilt angles of the transition dipole moments and of the hydrocarbon chains. (a) Au|dPC¦hPE, (b) Au|dPE¦hPC, (c) Au|hPE¦dPC, (d) Au|hPC¦dPE. Pink open squares: symmetric stretching, green open circles: asymmetric stretching, filled triangles: chain. The values of charge density for each potential are taken from the authors' data reported in *J. Electroanal. Chem.* **2018**, *819*, 338–346. Copyright © Elsevier 2017).[S1]

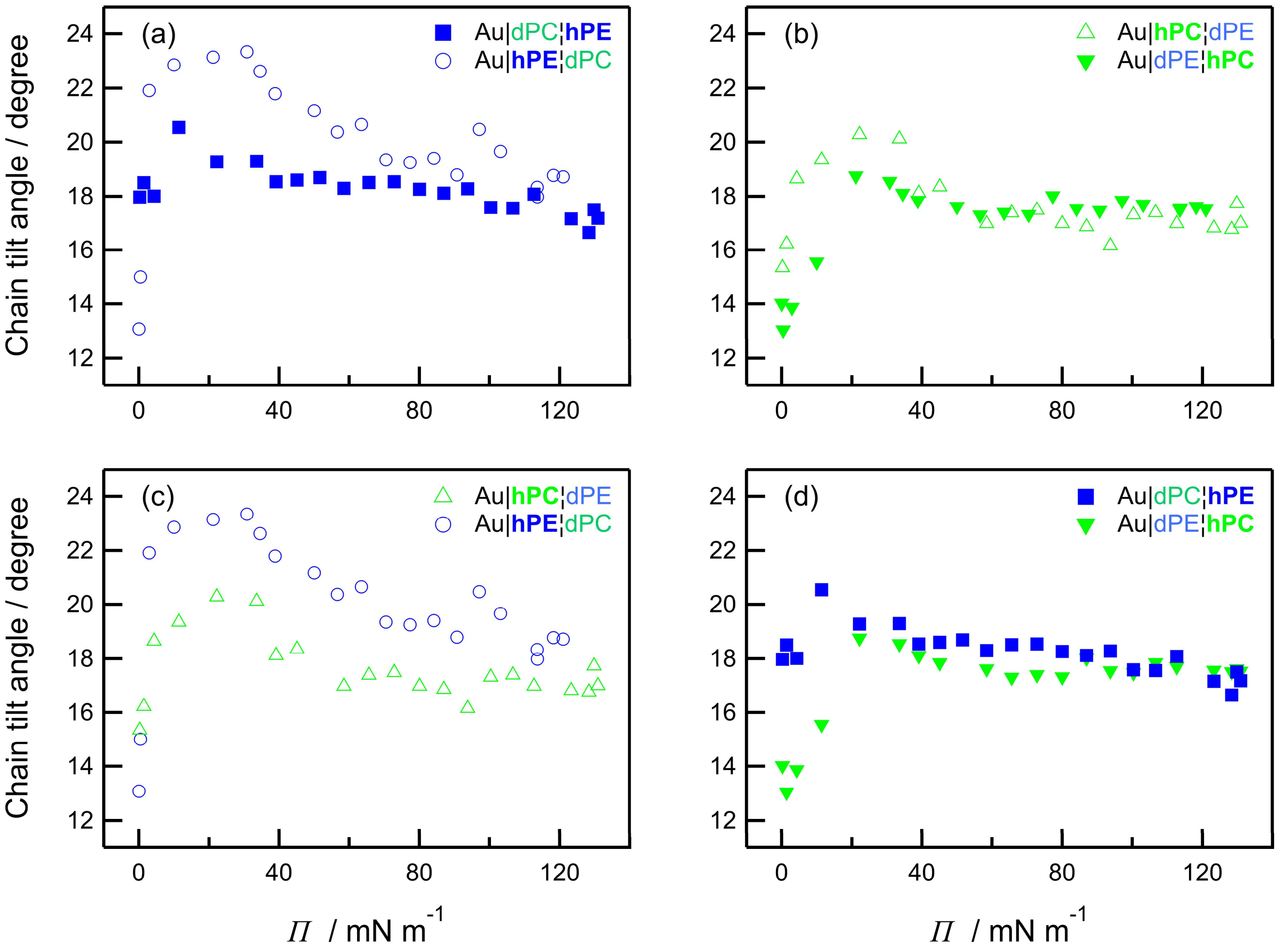

Figure S8 Comparisons of the dependence of chain tilt angles on surface pressure. (a) compares how DMPE behaves in each half of the bilayer. (b) compares how DMPC behaves in each half of the bilayer. (c) compares the behavior of different lipids in the electrode-facing monolayer. (d) compares the behavior of different lipids in the outer monolayer. The error bars have been omitted for clarity but are on the order of 4°. The values of surface pressure for each potential are calculated from the authors' data reported in *J. Electroanal. Chem.* **2018**, *819*, 338–346. Copyright © Elsevier 2017).[S1]

References

S1 Madrid, E.; Horswell, S. L. The Electrochemical Phase Behaviour of Chemically Asymmetric Lipid Bilayers Supported at Au(111) Electrodes. *J. Electroanal. Chem.* **2018**, *819*, 338–346.
